# Transport Characteristics and Parametric Sensitivity of a Single-Stage Circular-Channel Knudsen Pump

**DOI:** 10.3390/mi17080981

**Published:** 2026-08-20

**Authors:** Dingdong Zhang, Tongchao Zhao, Laixi Zhang, Marcos Rojas-Cárdenas, Stéphane Colin

**Affiliations:** 1 School of Mechanical and Electrical Engineering, Lanzhou University of Technology, Lanzhou 730050, China; 18663087263@163.com (T.Z.); laixi_zh@163.com (L.Z.); 2 Key Laboratory of Intelligent Integration Technology for Complete Equipment, Ministry of Education, Lanzhou University of Technology, Lanzhou 730050, China; 3 Engineering Research Center of New Equipment of Nonferrous Metallurgy, Ministry of Education, Lanzhou University of Technology, Lanzhou 730050, China; 4 Univ Toulouse, IMT Mines Albi, INSA Toulouse, ISAE-SUPAERO, CNRS, ICA, 31400 Toulouse, France; marcos.rojas@insa-toulouse.fr (M.R.-C.); stephane.colin@insa-toulouse.fr (S.C.)

**Keywords:** Knudsen pumps, thermal transpiration, rarefied gas dynamics, circular channels, linearized Shakhov model, parametric sensitivity

## Abstract

Thermal transpiration enables a Knudsen pump to transport gas without moving components. An axial-integration formulation based on pre-computed transport coefficients from the linearized Shakhov kinetic model is applied to a single-stage unit comprising a circular microchannel and a circular macrochannel in series, with opposite wall-temperature gradients. The pressure-generation and gas-transport capabilities are characterized by the maximum pressure difference or thermomolecular pressure difference (TPD), the maximum mass flow rate, the equivalent TPD, the equivalent flow resistance, and the complete mass-flow-rate–pressure-difference characteristics. The principal quantitative calculations cover temperature differences ranging from 10 to 50 K, while the 75 and 100 K cases are retained only to assess the persistence of the calculated trends. The formulation reproduces benchmark experimental TPD data with a maximum absolute relative deviation of 12.5% and a mean absolute relative deviation of 6.7%, and shows excellent agreement with numerical data from the literature, with deviation below 1%. A decomposition of the microchannel and macrochannel contributions shows that a macrochannel contributing little to the total equivalent flow resistance may nevertheless produce appreciable reverse thermal transpiration. At the baseline condition of the study and for an inlet pressure $P_{i}=10\;\mathrm{kPa}$, the macrochannel contributes only 0.37% of the total equivalent flow resistance but cancels 15.2% of the microchannel equivalent TPD. Over $P_{i}=1\text{–}50\;\mathrm{kPa}$, the temperature-difference sensitivity of the TPD ranges from 0.93 to 1.05, whereas the microchannel-radius sensitivity varies from −0.41 to −1.62. For the maximum mass flow rate, the microchannel-radius sensitivity ranges from 2.06 to 2.56 and the microchannel-length sensitivity remains close to −1, while the macrochannel-length effect is negligible. Increasing the macrochannel radius improves both limiting outputs, i.e., TPD and maximum mass flow rate, but with progressively diminishing benefits from further enlargement of the macrochannel. These results provide a quantitative basis for preliminary dimension selection while explicitly identifying the limitations associated with linearization, finite channel length, fully developed flow, and neglected interface losses.

## 1. Introduction

Thermal transpiration is a rarefied-gas transport phenomenon in which gas in contact with a wall subjected to a tangential temperature gradient moves from the colder region toward the hotter region. The appropriate conditions of rarefaction can be reached when the gas is flowing through micro- or nanoscale channels. A Knudsen micropump (KµP) exploits this effect to generate gas flow or a pressure difference without any moving mechanical components, enabling vibration-free operation, a potentially long service life, and compatibility with miniaturized systems. Wang et al. [1] reviewed the various physical mechanisms, structural configurations, fabrication methods and applications of KµPs. Wongwiwat et al. [2] demonstrated their potential as part of a hydrocarbon-fueled electrical-power-generation system, whereas Cheng et al. [3] developed a bidirectional pump using a three-dimensional printed thermal-management platform for microscale gas chromatography applications. Zhao et al. [4] further integrated three Knudsen pumps with a preconcentrator, separation column and detector on a single microscale gas-chromatography chip, illustrating the system-level advantages of motionless gas pumping. A major difficulty in designing KµPs is generating temperature gradients, which requires combining low-thermal-conductivity materials with heating elements and is therefore incompatible with microchannels etched in silicon. A technologically challenging solution to reduce heat conduction through the substrate is to suspend the microchannels. Byambadorj et al. [5] fabricated mechanically robust Silicon-On-Insulator (SOI) through-wafer pumps without fragile suspended pumping channels. As the level of rarefaction depends on both the channel cross-sectional dimensions and the operating pressure, microchannels generally provide appropriate rarefaction conditions only at pressures below atmospheric pressure. Recently, Basdanis et al. [6] proposed an original design based on nanochannels etched in glass using Bessel beam technology. This design can operate at atmospheric and above-atmospheric pressures.

KµPs are characterized by two performance metrics: the maximum pressure difference or thermomolecular pressure difference (TPD), obtained at zero mass flow rate, and the maximum mass flow rate, obtained at zero pressure difference. Over the last few years, considerable effort has been devoted to increase these metrics and manufacture functional KµPs. Increasing the TPD requires a multistage design. An et al. [7] developed a 162-stage silicon-micromachined pump for on-chip vacuum generation, while Kugimoto et al. [8] proposed a prediction method for multistage performance. Nakaye et al. [9] used multiple Knudsen-pump elements to construct a gas separator. An alternative to micro- or nanochannels is the use of porous media, which offer the advantage of a high channel density, but at the expense of a complex and poorly controlled pore geometry. Sugimoto et al. [10] analyzed the mechanism of thermal transpiration through porous media, while Parittothok et al. [11] improved the performance of glass microfiber pumps by incorporating triply periodic minimal surfaces. Kosyanchuk et al. [12] combined theoretical analysis with experiments on a 3D-printed pump incorporating active liquid heating and cooling. Multistage compression in rectangular channels was investigated by Zhao et al. [13], whereas Xiao et al. [14] examined a multistage configuration designed for low-temperature operation. These studies demonstrate that increasing the number of stages, modifying the porous structure, and improving thermal-management can enhance performance, at the expense of a more complex geometric design and thermal management strategy.

The effects of gas composition have also received increasing attention. Zhang et al. [15] investigated, using the Direct Simulation Monte Carlo (DSMC) method, the behavior of N_2_–O_2_ gas mixtures in KµPs with rectangular microchannels. Du et al. [16] also used DSMC to simulate the transport of H_2_–N_2_ mixtures in KµPs and compared the Variable Hard Sphere (VHS) and Variable Soft Sphere (VSS) molecular collision models. Yakunchikov and Kosyanchuk [17] numerically investigated species separation in noble gas mixtures transported through KµPs.

The channel geometry can also have a significant impact on KµP performance. Shao et al. [18] examined how obstacles inside a hydrogen Knudsen compressor affect pressure generation and local flow behavior. Ye et al. [19] analyzed multistage hydrogen compression driven by thermal transpiration, and their subsequent three-dimensional study [20] showed that the transverse channel geometry can alter both the internal flow field and pump performance. The influence of gradually varying outlet dimensions was recently quantified by Ye et al. [21]. From a reliability perspective, Lan et al. [22] studied the effects of the number and location of blocked microchannels resulting from microfabrication defects, whereas Huang et al. [23] investigated blockage in multistage hydrogen pumps.

The theoretical description of thermal transpiration has evolved from continuum approximations to kinetic formulations covering a broad range of rarefaction conditions. Méolans and Graur [24] derived a continuum analytical description of thermal creep and clarified the connection between wall-temperature gradients and induced gas motion. For long channels, Sharipov investigated non-isothermal flow in rectangular microchannels [25] and rarefied flow through long rectangular channels [26]. He also derived analytical solutions for circular tubes at arbitrary outlet-to-inlet temperature ratios [27] and for arbitrary inlet and outlet pressure and temperature differences [28]. Sharipov and Bertoldo [29] subsequently extended the long-tube treatment to channels of variable radius. The tabulated kinetic data compiled by Sharipov and Seleznev [30] provide an important reference for internal rarefied-gas transport. Experimental measurements by Rojas Cardenas et al. [31] provided TPD and mass flow rate data for a circular microtube subjected to a temperature gradient, establishing a valuable benchmark for circular-channel models. Kinetic simulations have enabled the analysis of geometries and operating conditions beyond the reach of analytical models. Sugimoto [32] performed quantitative kinetic calculations for a KµP, while Yamaguchi and Kikugawa [33] used molecular dynamics to examine the internal structure of thermal-transpiration flow fields. Tatsios et al. [34] investigated and parameterized pumping through long tapered channels. Rovenskaya [35] analyzed thermally generated flow between saw-tooth surfaces. López Quesada et al. [36] formulated application-oriented design guidelines for several pump architectures and later studied multistage assemblies containing converging, diverging, and uniform channels [37]. DSMC simulations have been applied to thermally induced forces around non-isothermal beams [38] and to rarefied flow through converging and diverging passages [39,40]. Recent advances in additive manufacturing have also enabled the experimental investigation of complex three-dimensional rarefied-flow structures, as demonstrated by Zhang et al. and Schweizer et al. [41,42].

Despite this broad body of work, one important question has not yet been analyzed in depth. A multistage KµP, required to achieve a significant TPD, connects the microchannels of one stage to those of the next through a larger channel, generally referred to as a macrochannel. In a stage consisting of a circular microchannel connected in series with a wider circular macrochannel, the temperature gradients have opposite directions in the two channels. The microchannel produces the primary positive thermal-transpiration effect, whereas the macrochannel generates an opposing thermal-transpiration flow while also contributing to the overall flow resistance. The extent to which these two effects independently influence the maximum pressure difference and the maximum flow rate has not been systematically quantified within a common parameter space. Furthermore, dimensional parametric curves alone do not provide a dimensionless comparison of the effects of temperature difference, microchannel radius, microchannel length, macrochannel radius, and macrochannel length, nor do they reveal how the relative importance of these parameters changes with inlet pressure.

This study applies an axial-integration formulation based on pre-computed transport coefficients of the linearized Shakhov kinetic model to a single-stage circular microchannel–macrochannel unit. The underlying kinetic formulation and locally fully developed-flow approximation follow established long-channel approaches; the contribution of this work lies in the systematic application of this framework to a circular microchannel–macrochannel unit, the quantitative decomposition of the thermal-driving and resistance contributions, and dimensionless sensitivity mapping. First, the model is validated against the circular-microtube thermomolecular-pressure data of Rojas-Cárdenas et al. [31] and the numerical mass-flow-rate results obtained using the method of Tantos [43]. The effects of temperature difference, microchannel radius, microchannel length, macrochannel radius, and macrochannel length are then evaluated through the TPD, the maximum mass flow rate, and the complete mass-flow-rate–pressure-difference characteristics. The net equivalent thermal-transpiration pressure is decomposed into positive microchannel and reverse macrochannel contributions, while the total equivalent resistance is decomposed into microchannel and macrochannel components. Finally, dimensionless logarithmic sensitivities are calculated at several inlet pressures to identify the parameters governing pressure generation and flow delivery. The investigated temperature and aspect-ratio ranges are restricted to reduce uncertainties associated with linearization and short-channel effects, and the limitations associated with the fully developed-flow assumption and the neglect of junction losses are explicitly discussed.

## 2. Theoretical Model and Calculation Method

### 2.1. Working Principle of a Single-Stage Knudsen Micropump

**Figure 1:**
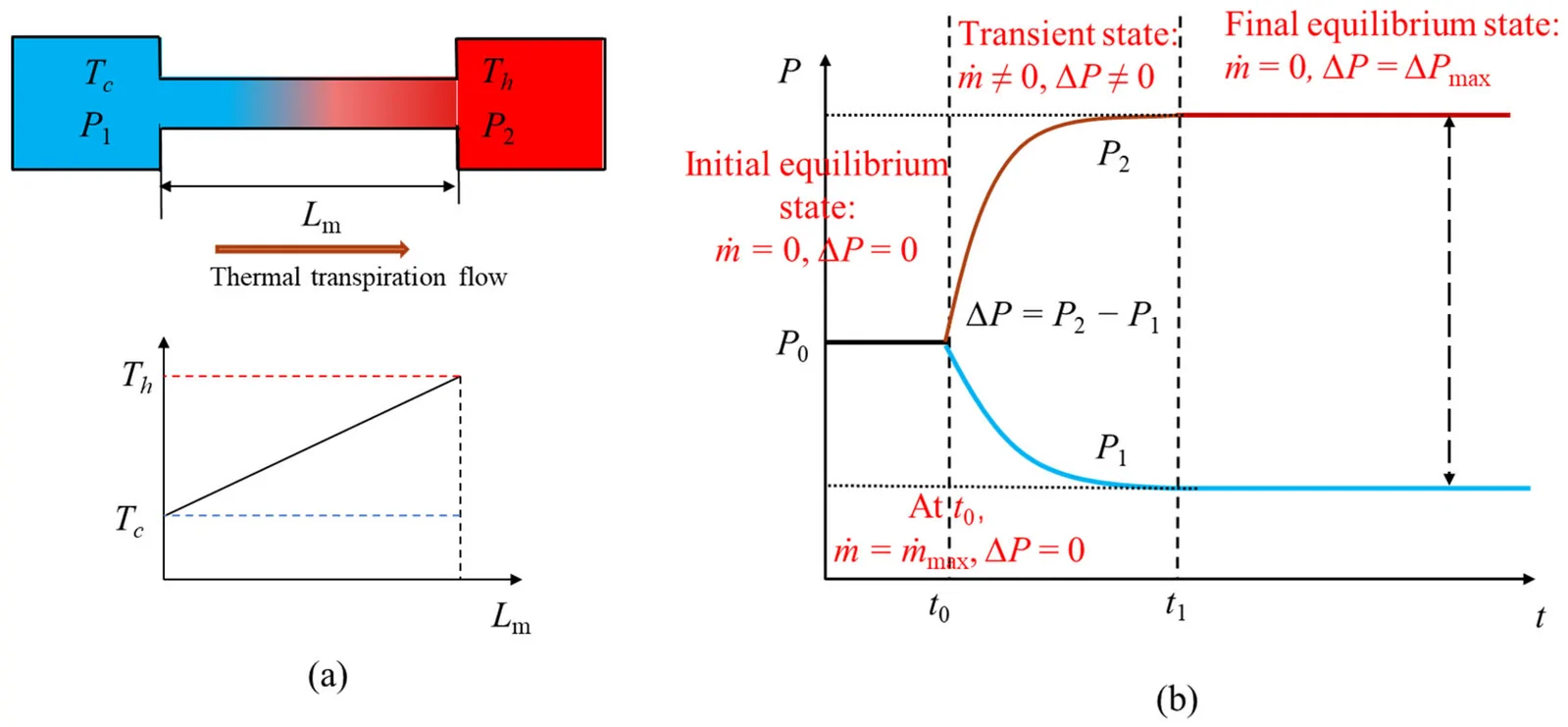
(**a**) Schematic of a classical single-stage KµP driven by thermal transpiration; (**b**) evolution of chamber pressures with time. In (**a**) Blue and red denote the cold and hot chambers, respectively, while the orange arrow indicates the direction of mass flow. In (**b**), the blue and red curves represent *P*_1_ and *P*_2_, respectively.

A classical single-stage KµP consists of two chambers connected by a microchannel. The chambers are maintained at different temperatures, $T_{h}$ and $T_{c}$, with $T_{h}>T_{c}$. A wall-temperature gradient directed from the cold side to the hot side induces, through nonequilibrium gas–wall interaction, a thermal-transpiration flow that drives gas from the cold side to the hot side, as shown in Figure 1a.

Initially, if the two chambers have the same pressure, $P_{1}=P_{2}$, thermal-transpiration first drives gas from the cold side to the hot side. This increases the pressure in the hot chamber and decreases the pressure in the cold chamber, forming a pressure difference $\Delta P=P_{2}-P_{1}$. As the pressure difference increases, pressure-driven backflow from the hot side to the cold side gradually develops. Eventually, the thermal transpiration flow and the pressure-driven backflow balance each other, the net mass flow rate becomes zero, and a steady pressure difference is established, as shown in Figure 1b. The TPD, defined as the maximum pressure difference $\Delta P_{\max}$, and the maximum mass flow rate ${\dot{m}}_{\max}$ are commonly used to characterize the performance of a Knudsen pump.

### 2.2. Typical Flow Conditions

**Figure 2:**
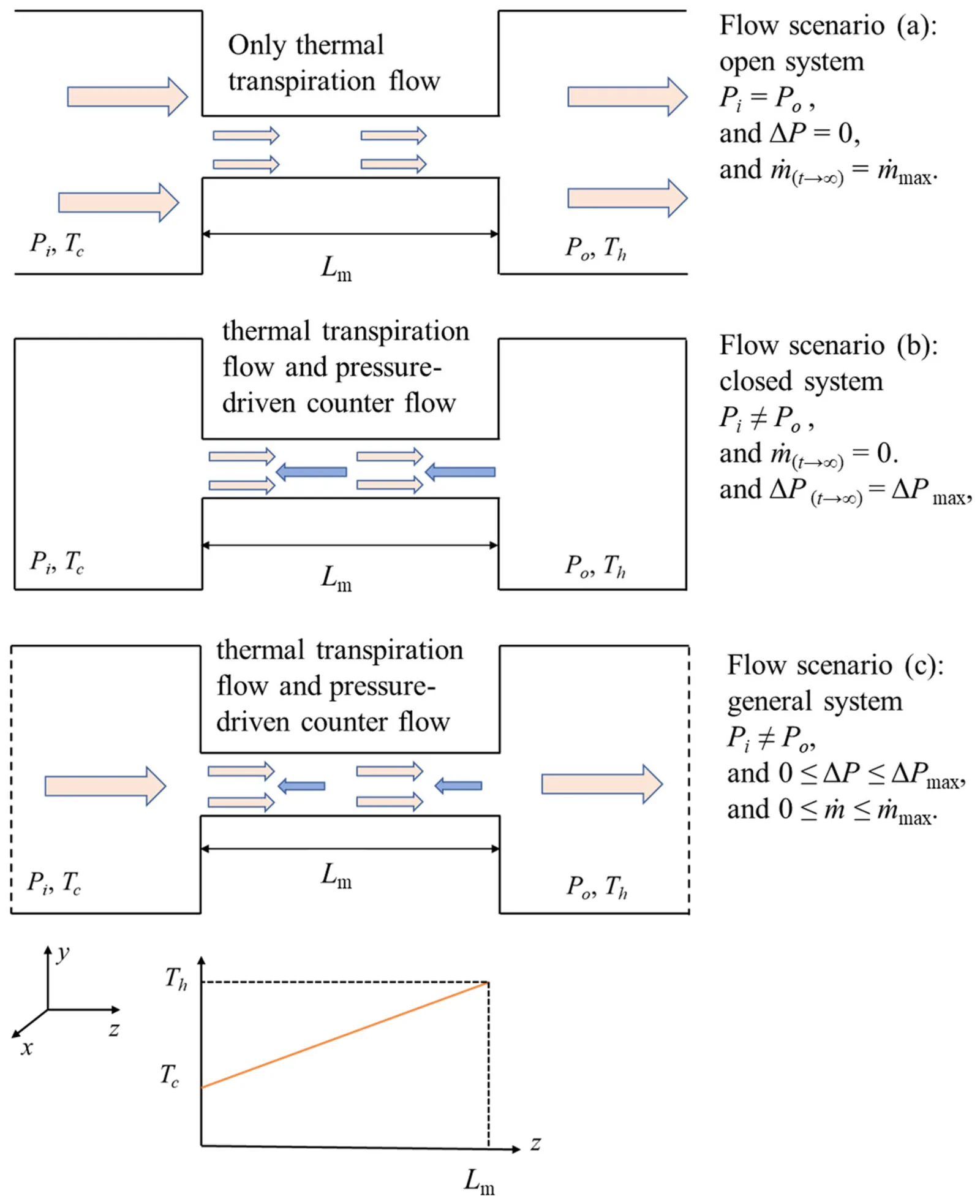
Basic unit of a KµP and different flow scenarios: (**a**) open-system, (**b**) closed-system, and (**c**) general operating configurations. The orange and blue arrows indicate the thermal transpiration flow and pressure-driven counter flow, respectively.

To analyze thermal transpiration in a Knudsen pump, three characteristic operating conditions are considered, as shown in Figure 2.

The first is the open-system configuration (Figure 2a). In this case, the pressures in the cold (or inlet) and hot (or outlet) chambers are equal, i.e., Δ*P* = *P_o_* − *P_i_* = 0, and the steady mass flow rate reaches its maximum value, denoted by ${\dot{m}}_{\max}$.

The second is the closed-system configuration (Figure 2b). Here, the net mass flow rate is zero, i.e., $\dot{m}=0$, and the corresponding steady pressure difference between the hot and cold chambers reaches its maximum value, denoted by Δ*P*_max_.

The third is the general operating configuration (Figure 2c), which lies between the open- and closed-system configurations. In this case, both thermal transpiration and pressure-driven backflow are present, resulting in a non-zero mass flow rate associated with a finite pressure difference between the hot and cold chambers. Practical Knudsen pumps generally operate under these conditions.

### 2.3. Structure Analyzed in the Present Work

**Figure 3:**
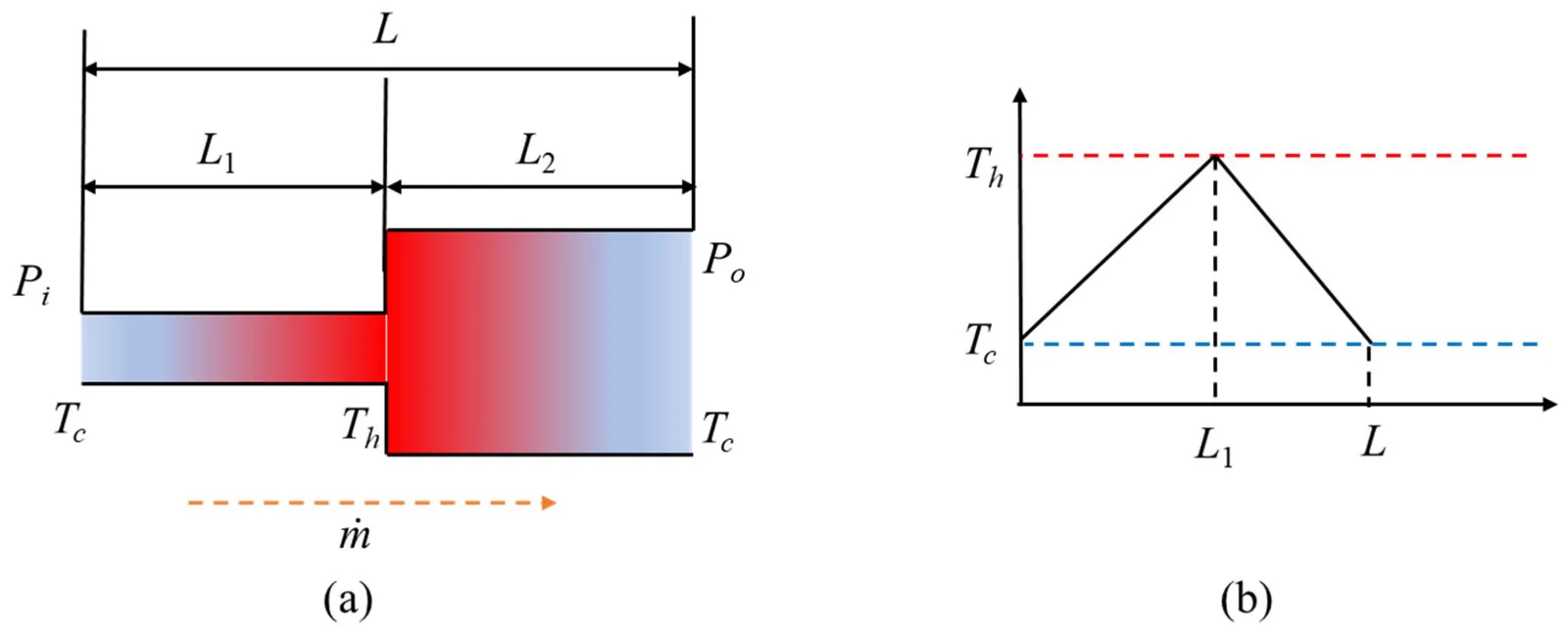
(**a**) Circular microchannel–macrochannel unit and (**b**) prescribed piecewise-linear wall-temperature distribution. Red and blue denote the hot and cold regions, respectively; while the orange dashed arrow in (**a**) indicates the flow direction, the red and blue dashed lines in (**b**) indicate the corresponding hot and cold wall temperatures.

The microchannel–macrochannel unit investigated in the present study is shown in Figure 3. It consists of a circular microchannel of radius *r* and length $L_{1}$, connected to a circular macrochannel of radius *R* and length $L_{2}$. The inlet and outlet are both maintained at temperature $T_{c}$, while the connection is maintained at $T_{h}$. The wall temperature therefore increases along the microchannel and decreases along the macrochannel.

The prescribed wall-temperature distribution is

(1) $$\begin{array}{l} T\left(z\right)=T_{c}+\frac{\Delta T}{L_{1}}z,\;0\leq z\leq L_{1}\\ T\left(z\right)=T_{h}-\frac{\Delta T}{L_{2}}\left(z-L_{1}\right),\;L_{1}\leq z\leq L_{1}+L_{2} \end{array}$$

where $\Delta T=T_{h}-T_{c}$. The local gas temperature entering the transport-coefficient formulation is assumed to be equal to the wall temperature given by Equation (1).

### 2.4. Local Mass Flow Rate Expression

For rarefied-gas flow in a long channel, the pressure-driven and thermally driven mass-flow components can be expressed by linear kinetic transport coefficients. In the present work, the channel is treated as a one-dimensional passage in which the pressure, temperature, and rarefaction parameter vary along the axial direction. At each axial location *z*, the flow is assumed to be locally represented by the transport-coefficient formulation for fully developed internal rarefied-gas flow. Following the approach used for long-tube rarefied-gas flows and thermally driven micropumps [36,37], the local mass flow rate is written as:

(2) $$\dot{m}=-\frac{A\left(z\right)L_{C}\left(z\right)}{v\left(z\right)}\left[G_{P}\left(\delta ,z\right)\frac{dP}{dz}-G_{T}\left(\delta ,z\right)\frac{P\left(z\right)}{T\left(z\right)}\frac{dT}{dz}\right]$$

where $\dot{m}$ is the mass flow rate, $P\left(z\right)$ is the pressure, $T\left(z\right)$ is the temperature, $A\left(z\right)$ is the channel cross-sectional area, $L_{C}\left(z\right)$ is a characteristic length of the channel cross-section, and $v\left(z\right)$ is the local most probable molecular speed. The pressure-driven and thermally driven transport coefficients, $G_{P}\left(\delta ,z\right)$ and $G_{T}\left(\delta ,z\right)$, were obtained from the tabulated dataset provided in the supplementary material in [44]. They depend on the local rarefaction parameter defined in Equation (5). The dataset was generated using the linearized Shakhov kinetic model with purely diffuse gas–surface reflection. The original normalization of the transport coefficients, the definition of the rarefaction parameter, and the tabulated range are reproduced here to ensure the complete traceability of the interpolation procedure. The first term in Equation (2) represents the pressure-driven mass-flow contribution, whereas the second term represents the thermally driven contribution (thermal transpiration).

The configuration considered in this study consists of a uniform circular microchannel connected in series to a circular macrochannel. Thus,

(3) $$A=\pi r_{C}^{2}$$

(4) $$L_{C}=r_{C}$$

where $r_{C}$ is the radius of the channel under consider ation, equal to *r* in the microchannel and to *R* in the macrochannel. The local rarefaction parameter is given by

(5) $$\delta \left(z\right)=\frac{P\left(z\right)L_{C}\left(z\right)}{\mu \left(z\right)v\left(z\right)}$$

where $\mu \left(z\right)$ is the dynamic viscosity. The temperature dependence of viscosity is given by

(6) $$\mu \left(z\right)=\mu_{ref}{\left(\frac{T\left(z\right)}{T_{ref}}\right)}^{\omega }$$

where $\mu_{ref}$ is the dynamic viscosity at the reference temperature $T_{ref}$, and *ω* is the viscosity exponent. This temperature dependence is consistent with the VHS and VSS molecular collision models. The local most probable molecular speed is given by

(7) $$v\left(z\right)=\sqrt{2R_{g}T\left(z\right)}$$

where $R_{g}$ is the specific gas constant. The thermophysical properties of helium, used as the working gas in this study, are listed in Table 1.

Equation (2) can be rearranged to obtain the axial pressure-gradient equation:

(8) $$\frac{dP}{dz}=-\frac{v\left(z\right)}{A\left(z\right)L_{C}\left(z\right)G_{P}\left(\delta ,z\right)}\dot{m}+\frac{G_{T}\left(\delta ,z\right)}{G_{P}\left(\delta ,z\right)}\frac{P\left(z\right)}{T\left(z\right)}\frac{dT}{dz}$$

The first term on the right-hand side represents the pressure gradient associated with viscous resistance, whereas the second term represents the pressure gradient generated by thermal transpiration. These two terms will subsequently be referred to as the resistance and thermal-driving contributions, respectively.

### 2.5. Axial Integration Method

In this study, the full molecular velocity distribution function is not solved for each operating condition. Instead, a calculation method based on the transport coefficients of the linearized Shakhov model is adopted. At each axial location, the local rarefaction parameter $\delta \left(z\right)$ is first evaluated from Equation (5). The corresponding $G_{P}$ and $G_{T}$ values are then obtained by interpolation of the tabulated transport coefficients reported in [44] and substituted into Equation (8) for axial integration. The tabulated rarefaction range used in the calculations is $0\leq \delta_{i}\leq 50$. At each axial location, the local value of δ is checked against the tabulated range before selecting the appropriate interpolation or asymptotic treatment.

When the local rarefaction parameter lies within the tabulated range, i.e., $\delta_{j}\leq \delta_{i}\leq \delta_{j+1}$, linear interpolation is applied:

(9) $$G_{P}\left(\delta_{i}\right)=G_{P}\left(\delta_{j}\right)+\frac{\delta_{i}-\delta_{j}}{\delta_{j+1}-\delta_{j}}\left[G_{P}\left(\delta_{j+1}\right)-G_{P}\left(\delta_{j}\right)\right]$$

(10) $$G_{T}\left(\delta_{i}\right)=G_{T}\left(\delta_{j}\right)+\frac{\delta_{i}-\delta_{j}}{\delta_{j+1}-\delta_{j}}\left[G_{T}\left(\delta_{j+1}\right)-G_{T}\left(\delta_{j}\right)\right]$$

If the rarefaction parameter exceeds the upper limit of the tabulated data, the corresponding large-δ asymptotic expression is used.

The microchannel–macrochannel unit is discretized with $4\times 10^{4}$ nodes per channel along the axial direction. For a given mass flow rate $\dot{m}$, Equation (8) is integrated from the inlet pressure $P_{i}$ to obtain the outlet pressure $P_{o}$. In the open-system configuration, the inlet and outlet pressures are equal. The mass flow rate is iterated until ΔP=0 is satisfied, and the corresponding value of $\dot{m}$ is the maximum mass flow rate, ${\dot{m}}_{\max}$. In the closed-system configuration, $\dot{m}=0$ is imposed, and axial integration yields the maximum pressure difference, $\Delta P_{\max}$. In the general operating configuration, either $\dot{m}$ or $P_{o}$ is prescribed to obtain the corresponding mass-flow-rate–pressure-difference characteristic curve.

### 2.6. Equivalent Thermomolecular Pressure Difference and Flow Resistance

Integrating Equation (8) along the entire microchannel–macrochannel unit length, i.e., over 0≤z≤L where $L=L_{1}+L_{2}$, gives

(11) $$\Delta P=P_{o}-P_{i}=-\dot{m}\int_{0}^{L}\frac{v\left(z\right)}{A\left(z\right)L_{C}\left(z\right)G_{P}\left(\delta ,z\right)}dz+\int_{0}^{L}\frac{G_{T}\left(\delta ,z\right)}{G_{P}\left(\delta ,z\right)}\frac{P\left(z\right)}{T\left(z\right)}\frac{dT}{dz}dz$$

The equivalent flow resistance is defined as

(12) $$R_{f}=\int_{0}^{L}\frac{v\left(z\right)}{A\left(z\right)L_{C}\left(z\right)G_{P}\left(\delta ,z\right)}dz$$

The equivalent thermomolecular pressure difference is defined as

(13) $$\Delta P_{T}=\int_{0}^{L}\frac{G_{T}\left(\delta ,z\right)}{G_{P}\left(\delta ,z\right)}\frac{P\left(z\right)}{T\left(z\right)}\frac{dT}{dz}dz$$

Thus, Equation (11) can be written in the compact form

(14) $$\dot{m}=\frac{\Delta P_{T}-\Delta P}{R_{f}}$$

Equation (14) expresses the mass flow rate generated by the KµP as the result of the balance between the equivalent thermomolecular pressure difference, the pressure difference between the inlet and outlet, and the equivalent flow resistance. It should be emphasized that $\Delta P_{T}$ and $R_{f}$ are evaluated from the axial pressure and temperature distributions along the two channels. They are therefore operating-point-dependent quantities rather than universal constants determined solely by the channel geometry.

For the open-system configuration, ΔP=0, and the corresponding maximum mass flow rate is

(15) $${\dot{m}}_{\max}=\frac{\Delta P_{T}^{o}}{R_{f}^{o}}$$

where the superscript *o* denotes quantities evaluated from the open-system solution.

For the closed-system configuration, $\dot{m}=0$, and Equation (14) therefore gives the maximum pressure difference as

(16) $$\Delta P_{\max}=\Delta P_{T}^{c}$$

where the superscript *c* denotes quantities evaluated from the closed-system solution. The distinction between the open- and closed-system quantities is necessary because the two operating conditions lead to different axial pressure distributions and, consequently, to different local rarefaction parameters and transport coefficients.

For the serial microchannel–macrochannel configuration shown in Figure 3, the equivalent thermomolecular pressure difference can be separated into two contributions:

(17) $$\Delta P_{T}=\Delta P_{T,micro}+\Delta P_{T,macro}$$

where the microchannel contribution is

(18) $$\Delta P_{T,micro}=\int_{0}^{L_{1}}\frac{G_{T}\left(\delta ,z\right)}{G_{P}\left(\delta ,z\right)}\frac{P\left(z\right)}{T\left(z\right)}\frac{dT}{dz}dz$$

and the macrochannel contribution is

(19) $$\Delta P_{T,macro}=\int_{L_{1}}^{L}\frac{G_{T}\left(\delta ,z\right)}{G_{P}\left(\delta ,z\right)}\frac{P\left(z\right)}{T\left(z\right)}\frac{dT}{dz}dz$$

For the temperature distribution considered in the present pump, the wall temperature increases from $T_{c}$ to $T_{h}$ along the microchannel and decreases from $T_{h}$ to $T_{c}$ along the macrochannel. Thus, the microchannel produces a positive equivalent TPD contribution, $\Delta P_{T,micro}>0$, whereas the macrochannel produces an opposing contribution, $\Delta P_{T,macro}<0$. The macrochannel therefore affects the pump through reverse thermal transpiration in addition to its contribution to the equivalent flow resistance.

This total equivalent flow resistance can likewise be decomposed into the contributions of the two channels:

(20) $$R_{f}=R_{f,micro}+R_{f,macro}$$

where the microchannel contribution is

(21) $$R_{f,micro}=\int_{0}^{L_{1}}\frac{v\left(z\right)}{A\left(z\right)L_{C}\left(z\right)G_{P}\left(\delta ,z\right)}dz$$

and the macrochannel contribution is

(22) $$R_{f,macro}=\int_{L_{1}}^{L}\frac{v\left(z\right)}{A\left(z\right)L_{C}\left(z\right)G_{P}\left(\delta ,z\right)}dz$$

Although the two channels provide different equivalent TPD and flow-resistance contributions, the mass flow rate is identical in the microchannel and macrochannel. Therefore, the quantities defined in Equations (18) and (19) and Equations (21) and (22) should be interpreted as coupled contributions obtained from the overall operating-point solution, rather than as independent properties of the two channels.

To quantify the reduction in the equivalent thermomolecular pressure difference generated by the microchannel due to the opposing contribution of the macrochannel, an equivalent TPD ratio is defined as

(23) $$\chi_{T}=-\frac{\Delta P_{T,macro}}{\Delta P_{T,micro}}$$

which is positive because the microchannel and macrochannel contributions have opposite signs. In addition, the fraction of the total flow resistance contributed by the macrochannel is defined as

(24) $$\chi_{R}=\frac{R_{f,macro}}{R_{f}}$$

The two indicators, $\chi_{T}$ and $\chi_{R}$, describe different physical mechanisms. The former quantifies the reduction in the equivalent TPD due to the opposing macrochannel contribution, with $\chi_{T}=0$ corresponding to no reduction and $\chi_{T}=1$ to complete cancelation of the microchannel contribution, whereas the latter quantifies the macrochannel share of the total flow resistance.

If the variations of $R_{f}$ and $\Delta P_{T}$ between the open- and closed-system operating conditions are small relative to their mean values, Equation (14) gives the following approximate normalized characteristic relation:

(25) $$\frac{\dot{m}}{{\dot{m}}_{\max}}\approx 1-\frac{\Delta P}{\Delta P_{\max}}$$

Equation (25) explains the approximately linear mass-flow-rate–pressure-difference curves obtained for many of the investigated conditions. It should not, however, be interpreted as an exact constitutive relation. As the operating point changes, the axial pressure distribution, local rarefaction parameter, and transport coefficients are updated. Consequently, $R_{f}$ and $\Delta P_{T}$ may vary along the characteristic curve, resulting in small deviations from strict linearity.

### 2.7. Dimensionless Local Sensitivity

Direct comparison of the absolute variations caused by different parameters is not sufficient because the temperature difference, radii, and lengths have different physical dimensions and characteristic magnitudes. A dimensionless logarithmic sensitivity coefficient is therefore introduced. For an output quantity $Y\in \left\{\Delta P_{\max},{\dot{m}}_{\max}\right\}$ and an input parameter $X\in \left\{\Delta T,r,R,L_{1},L_{2}\right\}$, the local logarithmic sensitivity is defined as

(26) $$S_{X}^{Y}={\left.\frac{\partial \ln Y}{\partial \ln X}\right|}_{X=X_{0}}={\left.\frac{X}{Y}\frac{\partial Y}{\partial X}\right|}_{X=X_{0}}$$

where $X_{0}$ is the reference value of the selected parameter. In the numerical calculations, the sensitivity is evaluated using a centered logarithmic difference:

(27) $$S_{X}^{Y}\approx \frac{\ln Y\left[\left(1+\varepsilon \right)X_{0}\right]-\ln Y\left[\left(1-\varepsilon \right)X_{0}\right]}{\ln\left(1+\varepsilon \right)-\ln\left(1-\varepsilon \right)}$$

Thus, by choosing for example ε=0.1, each parameter *X* is perturbed by ±10% about its reference value, while the remaining dimensional parameters and inlet pressure are held fixed. A positive sensitivity indicates that increasing the parameter increases the selected output, whereas a negative sensitivity indicates an inverse effect. The magnitude $\left|S_{X}^{Y}\right|$ measures the strength of the local response. Additional calculations with ε=0.05 and 0.20 are performed for representative conditions to verify that the resulting parameter ranking is not an artifact of the selected perturbation interval.

### 2.8. Numerical Checks and Model Applicability

**Figure 4:**
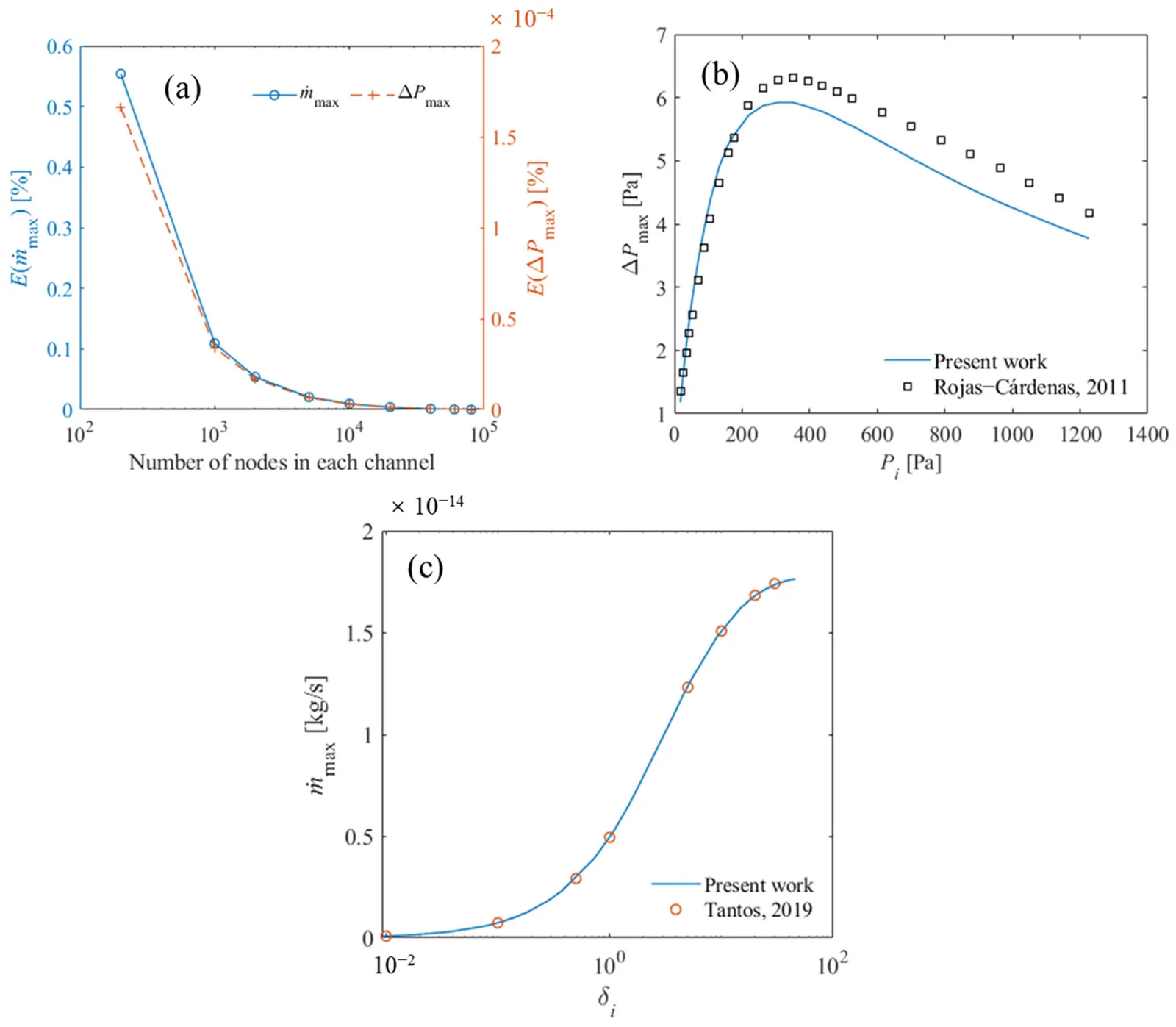
Numerical checks and benchmark comparisons. (**a**) Relative axial–discretization errors of ∆*P*_max_ and $\dot{m}$_max_; (**b**) comparison with the experimental data of Rojas-Cárdenas et al. [31]; (**c**) comparison with the numerical results obtained using the method of Tantos [43].

The numerical reliability of the axial-integration procedure was first examined through an axial-discretization independence test. The baseline configuration was $T_{c}=300\;K$, $T_{h}=350\;K$, *r* = 5 μm, *R* = 25 μm, and *L*_1_ = *L*_2_ = 500 μm and $P_{i}=10\;\mathrm{kPa}$. The number of axial nodes in each channel was varied from $N=2\times 10^{2}$ to $N=N_{ref}=8\times 10^{4}$. Taking the $N_{ref}$-node results as the reference, the relative error was evaluated as

(28) $$E_{N}\left(Y\right)=\left|\frac{Y_{N}-Y_{N_{ref}}}{Y_{N_{ref}}}\right|$$

where *Y* represents $\Delta P_{\max}$ or ${\dot{m}}_{\max}$. As shown in Figure 4a, both outputs became insensitive to further mesh refinement when $N\geq 4\times 10^{4}$. The corresponding errors were 0.001% and 0.00001%, respectively; therefore, $4\times 10^{4}$ nodes per channel were used in the following calculations.

The circular-microtube experiment of Rojas Cardenas et al. [31] was used as an independent benchmark for the long-channel transport formulation. The selected dataset corresponds to helium with an imposed temperature difference of 50 K and inlet pressures ranging from 17.51 Pa to 1225.97 Pa, as reported in Figure 4a of Ref. [31]. The experimental geometry, thermal conditions, operating mode, and corresponding assumptions used in the present reconstruction are summarized in Table 2. Because the original study described the cold side as being maintained at room temperature without specifying a unique numerical value, $T_{c}=292\;K$ was adopted as a representative reconstruction value and $T_{h}=342\;K$ was prescribed.

The reference study reported the thermomolecular pressure difference as a function of a representative rarefaction parameter. To use the present pressure-controlled solver, the corresponding rarefaction coordinates were represented by 27 equivalent inlet pressures using the same helium viscosity law and temperature conditions as those employed in the present calculation. For each pressure, a single uniform tube was modeled and the zero-net-flow condition was imposed. Although only one tube was considered, the opposing pressure-driven contribution was retained implicitly through the resulting pressure gradient.

The pointwise absolute relative deviation was calculated as

(29) $$E_{i}=\left|\frac{\Delta P_{cal,i}-\Delta P_{\exp,i}}{\Delta P_{\exp,i}}\right|$$

where $\Delta P_{cal,i}$ is the TPD calculated in the present study and $\Delta P_{\exp,i}$ is the corresponding experimental value reported in Ref. [31]. The mean absolute relative deviation was obtained from

(30) $$E_{mean}=\frac{1}{N_{d}}\sum_{i=1}^{N_{d}}E_{i}$$

with $N_{d}=27$ compared data points. As shown in Figure 4b, the present model reproduces the experimentally observed increase, maximum, and subsequent decline of the thermomolecular pressure difference with increasing rarefaction. The maximum pointwise absolute relative deviation is $E_{i,\max}=12.5\%$, and the mean absolute relative deviation is $E_{mean}=6.7\%$. These deviations may result from experimental uncertainties, the representative room-temperature assumption, and the fully developed, one-dimensional, and fully diffuse gas–wall interaction assumptions adopted in the model. The agreement is considered reasonable for assessing the circular-tube transport coefficients and the axial-integration procedure. However, because the experimental data concern a single microchannel, this comparison does not provide information on possible local effects at the microchannel–macrochannel interface.

It should be noted that the linearized Shakhov formulation is formally associated with small disturbances from equilibrium. Accordingly, the results obtained for $\Delta T=T_{h}-T_{c}\leq 50\;K$ are considered the principal quantitatively relevant results of the present study. The cases with ΔT=75 K and 100 K are additionally investigated to assess whether the observed trends persist at larger temperature differences. These cases are intended as exploratory calculations and should not be interpreted as independently validated predictions under strongly nonequilibrium conditions.

However, Figure 4c compares the present results with the numerical data based on the Rykov model reported by Tantos [43] for helium modeled as a hard-sphere gas flowing through a microtube with a radius *r* = 0.4 μm, a length *L* = 500 μm, and temperatures $T_{h}=600\;K$ and $T_{c}=300\;K$. The maximum mass flow rate, ${\dot{m}}_{\max}$, is plotted as a function of the inlet rarefaction parameter, $\delta_{i}$, evaluated using the inlet pressure and temperature. Excellent agreement is obtained, with the deviation between the two sets of data remaining below 1%. Because the temperature ratio is $T_{h}/T_{c}=2$, this benchmark is used primarily as a numerical cross-check of the transport-coefficient interpolation, dimensional conversion, and zero-pressure-difference simulation under the specific conditions of Ref. [43], which involve a relatively large temperature ratio. The agreement should not be interpreted as a general validation of the linearized formulation for arbitrary large temperature ratios.

The model assumes locally fully developed flow in each cylindrical channel. The principal geometric cases therefore satisfy $L_{1}/r\geq 20$, and $L_{2}/R\geq 10$, which respects the recommendation $L_{C}/r_{C}\geq 10$ from Valougeorgis et al. [45]. These values are conservative screening criteria rather than universal thresholds. Entrance and exit regions, finite reservoirs, multidimensional nonequilibrium effects at the channel junction, abrupt expansion and contraction losses, wall roughness, and conjugate heat transfer are not resolved. Their omission is expected to affect finite-flow predictions more strongly, although local nonequilibrium may also modify the zero-flow thermomolecular pressure difference.

Extension of the calculated results to other gases or dimensions requires comparable values of the local rarefaction parameter, normalized temperature difference, geometric aspect ratios, radius ratio, and gas–surface accommodation condition. The reported results should therefore be interpreted as quantitative predictions within the stated ranges of gas properties, temperature difference, radii, and channel lengths.

## 3. Results and Discussion

Helium is considered as the working gas throughout. The baseline geometry is defined by *r* = 5 μm, *R* = 25 μm, and *L*_1_ = *L*_2_ = 500 μm, corresponding to $L_{1}/r=100$, R/r=5 and $L_{2}/R=20$. The cold-end temperature is $T_{c}=300\;K$ and the baseline temperature difference is ΔT=50 K. The inlet pressure $P_{i}$ ranges from 0.1 to 100 kPa. The ranges of the parameters investigated are summarized in Table 3.

To quantify the magnitude of the imposed temperature difference, a normalized temperature difference is defined as $\varepsilon_{T}=\frac{2\Delta T}{T_{h}+T_{c}}$. For $T_{c}=300\;K$ and ΔT=10, 20, 50, 75, and 100 K, the corresponding values of $\varepsilon_{T}$ are 0.033, 0.065, 0.154, 0.222, and 0.286, respectively.

### 3.1. Effect of Temperature Difference

**Figure 5:**
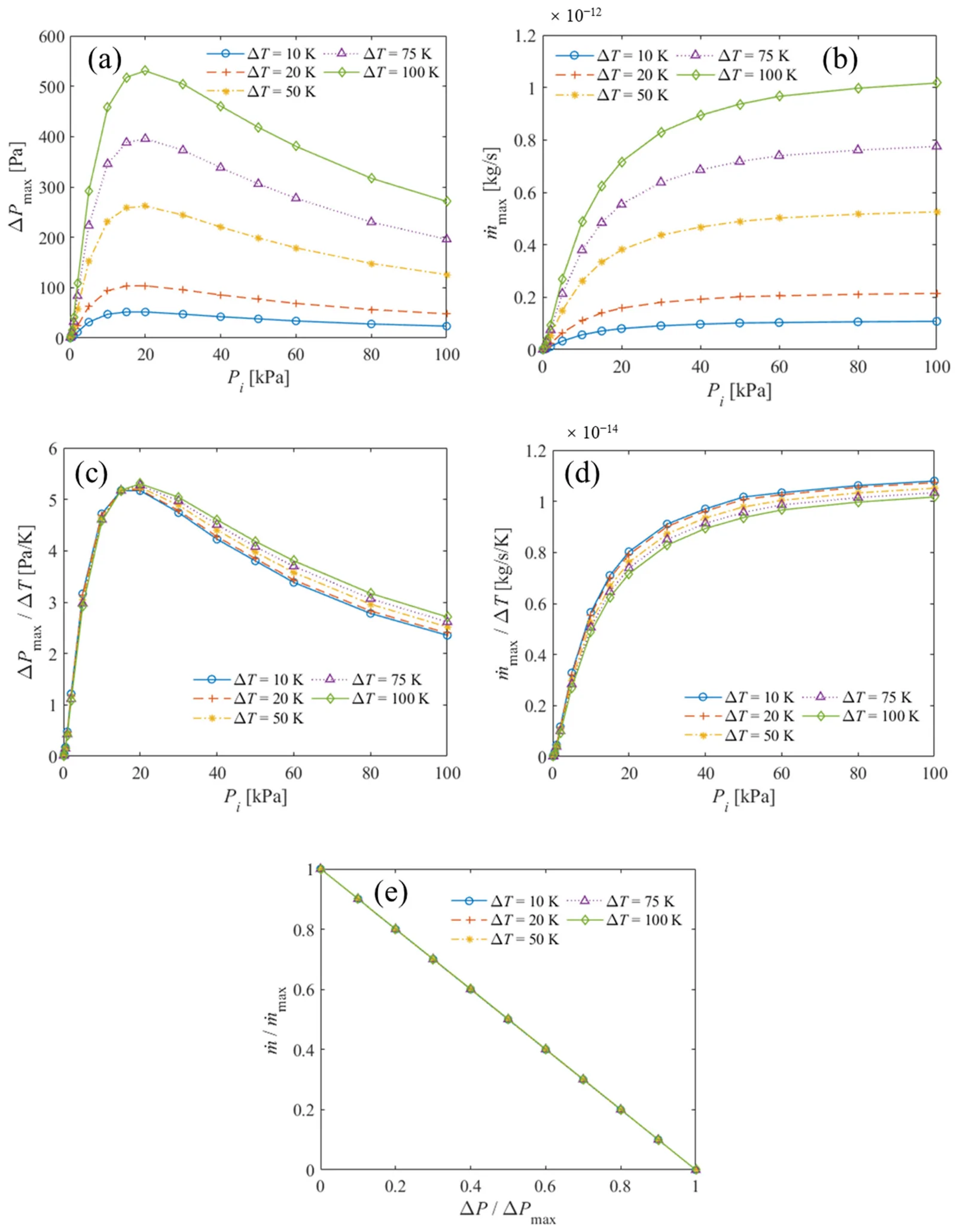
Effects of temperature difference on the KµP performance: (**a**) Δ*P*_max_; (**b**) $\dot{m}$_max_; (**c**) Δ*P*_max_/∆*T*; (**d**) $\dot{m}$_max_/∆*T*; and (**e**) normalized characteristic curves at *P_i_* = 10 kPa.

Figure 5 shows the effects of the imposed temperature difference ΔT on the TPD, maximum mass flow rate, normalized output quantities and characteristic curves. The calculations were performed for *r* = 5 μm, *R* = 25 μm, and *L*_1_ = *L*_2_ = 500 μm, and $T_{c}=300\;K$. The cases with ΔT=10, 20, and 50 K form the principal quantitative dataset. The 75 and 100 K cases are shown using the same formulation to assess whether the calculated trends and normalized characteristic behavior persist as the imposed temperature difference increases. Quantitative engineering predictions at these larger temperature differences would require nonlinear kinetic or experimental validation.

As shown in Figure 5a, the maximum pressure difference increases consistently with the imposed temperature difference over the complete inlet-pressure range. In the closed system, the net mass flow rate is zero, and the TPD equals the equivalent TPD. Increasing Δ*T* directly increases the imposed wall-temperature gradient and thereby strengthens the thermally driven contribution. For every temperature difference, $\Delta P_{\max}$ first increases with $P_{i}$, reaches a maximum at an intermediate pressure, and subsequently decreases. The non-monotonic pressure dependence results from the combined variation in the density-related factor P/T and the rarefaction-dependent transport-coefficient ratio $G_{T}/G_{P}$. At low pressures ($P_{i}<15\;\mathrm{kPa}$), the increase in gas density is dominant, whereas at higher pressures ($P_{i}>20\;\mathrm{kPa}$), the decrease in the transport-coefficient ratio $G_{T}/G_{P}$ associated with decreasing rarefaction reduces the relative thermal-transpiration contribution. The pressure corresponding to the maximum remains close to 15–20 kPa for the investigated temperature differences. This behavior indicates that increasing Δ*T* primarily increases the magnitude of the TPD without significantly shifting the rarefaction condition associated with the generation of the peak pressure difference.

Figure 5b shows that the maximum mass flow rate also increases with the temperature difference. Unlike $\Delta P_{\max}$, ${\dot{m}}_{\max}$ increases rapidly at low and intermediate inlet pressures and approaches a weakly pressure-dependent plateau at higher pressures. This result follows from Equation (15), because increasing the temperature difference enhances the equivalent TPD while the equivalent resistance variation remains limited.

The normalized quantities $\Delta P_{\max}/\Delta T$ and ${\dot{m}}_{\max}/\Delta T$ in Figure 5c,d allow a more direct assessment of the proportionality between pump output and temperature difference. The $\Delta P_{\max}/\Delta T$ curves remain close to each other, indicating an approximately proportional pressure response over the investigated range. However, the curves do not collapse exactly, particularly near and above the pressure corresponding to peak performance. The deviation from exact proportionality results from the temperature dependence of viscosity and molecular speed, together with the corresponding variations in the local rarefaction parameter and transport coefficients along the channels.

A somewhat stronger dependence on the temperature difference is observed for ${\dot{m}}_{\max}/\Delta T$ in Figure 5d. The normalized maximum mass flow rate decreases slightly as Δ*T* increases, especially at intermediate and high inlet pressures. Thus, the increase in maximum mass flow rate is slightly sublinear with respect to the imposed temperature difference. This behavior reflects the fact that the maximum mass flow rate is affected not only by the equivalent TPD but also by the temperature-dependent equivalent flow resistance.

Figure 5e presents the normalized characteristic curves at $P_{i}=10\;\mathrm{kPa}$. The curves for all temperature differences nearly collapse onto a single straight line, as expected from Equation (25). Therefore, within the present parameter range, changing the temperature difference primarily modifies the two limiting outputs but has little influence on the normalized shape of the characteristic curve. This result indicates that the complete operating characteristic can be reconstructed from ${\dot{m}}_{\max}$ and $\Delta P_{\max}$ for preliminary design purposes.

### 3.2. Decomposition of Equivalent TPD and Flow Resistance

**Figure 6:**
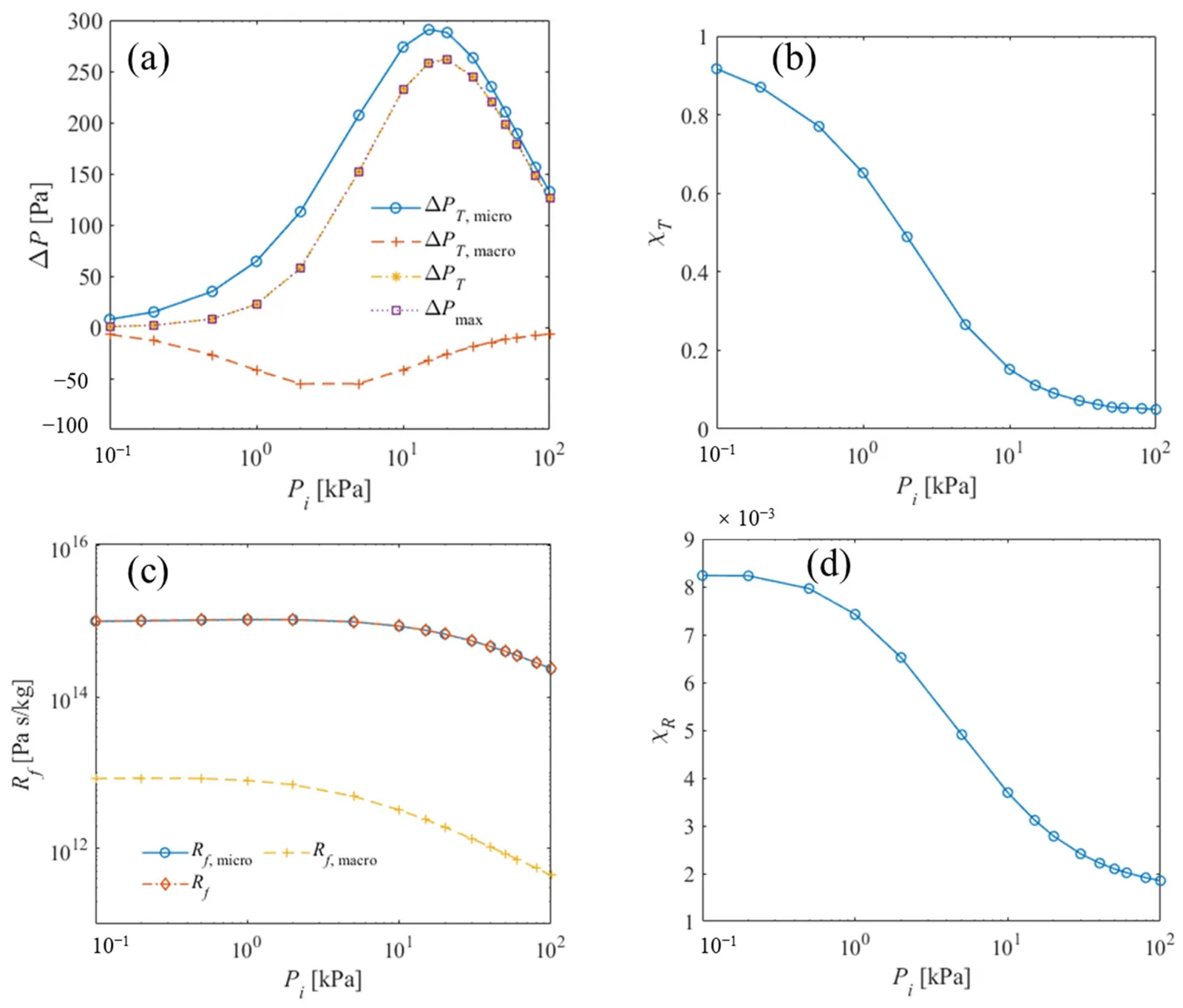
Decomposition of the equivalent TPD and flow resistance for the baseline configuration as a function of inlet pressure: (**a**) microchannel, macrochannel and net equivalent TPD; (**b**) equivalent TPD ratio; (**c**) microchannel, macrochannel and total equivalent flow resistances; and (**d**) fraction of the total flow resistance contributed by the macrochannel.

Figure 6 presents the microchannel and macrochannel contributions to the equivalent TPD and equivalent flow resistance for the baseline geometry in the open-system configuration. As shown in Figure 6a, the microchannel produces a positive equivalent TPD contribution, $\Delta P_{T,micro}$, whereas the reversed wall-temperature gradient in the macrochannel produces a negative contribution, $\Delta P_{T,macro}$. Their sum agrees with the directly calculated maximum pressure difference over the complete inlet-pressure range, i.e., $\Delta P_{\max}=\Delta P_{T}$, as expected for the zero-flow condition.

The equivalent TPD ratio plotted in Figure 6b decreases markedly with increasing inlet pressure. At the lowest pressure, the magnitude of the reverse macrochannel contribution is approximately 92% of the positive microchannel contribution. The ratio subsequently decreases to approximately 5% at $P_{i}=100\;\mathrm{kPa}$. Thus, the macrochannel strongly suppresses the net thermal driving under highly rarefied conditions, whereas its thermal contribution becomes relatively weak at moderate and high pressures, where the macrochannel operates under a lower degree of rarefaction.

In contrast, Figure 6c,d show that the macrochannel accounts for less than 1% of the total equivalent resistance throughout the investigated pressure range. The total resistance therefore nearly coincides with the microchannel resistance. This difference between the thermal and resistive contributions is important: a section with negligible flow resistance may still exert a substantial reverse thermal-transpiration effect. Accordingly, the macrochannel cannot be treated as a purely passive connection based on its resistance contribution alone.

At the baseline inlet pressure $P_{i}=10\;\mathrm{kPa}$, the macrochannel contributes only 0.37% of the total equivalent distributed resistance, but its reverse thermal-transpiration contribution cancels 15.2% of the positive microchannel equivalent TPD. This comparison demonstrates that the thermal importance of a channel section cannot be inferred from its resistance contribution alone.

The equivalent resistance decreases at moderate and high inlet pressures. Consequently, although the net equivalent TPD decreases beyond its peak, the simultaneous decrease in resistance allows the maximum mass flow rate to continue increasing before approaching a plateau. The mass flow rate reconstructed from the decomposed TPD and resistance contributions agrees with the directly calculated value, confirming the numerical consistency of the decomposition.

### 3.3. Effect of Microchannel Radius

**Figure 7:**
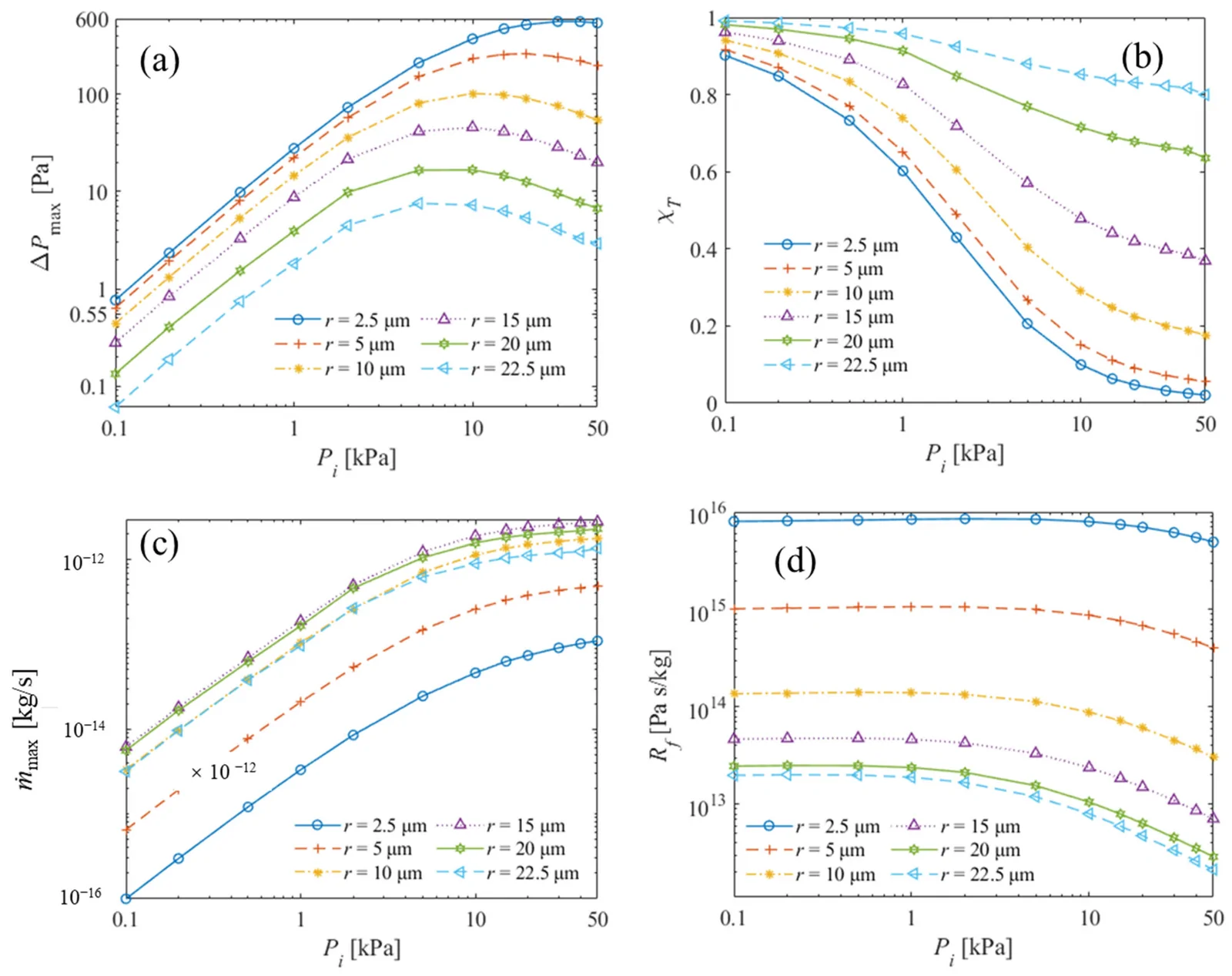
Effects of microchannel radius on (**a**) the TPD, (**b**) the equivalent TPD ratio, (**c**) the maximum mass flow rate, and (**d**) the equivalent flow resistance.

Figure 7 shows the effects of the microchannel radius on the TPD, equivalent TPD ratio, maximum mass flow rate and equivalent flow resistance. The macrochannel radius and the two channel lengths were fixed at *R* = 25 μm and *L*_1_ = *L*_2_ = 500 μm. To ensure consistency with the validated range of the transport-coefficient database, only inlet-pressure cases satisfying $\delta_{i}\leq 50$ are retained in Figure 7. Because $\delta_{i}$ depends on both pressure and channel radius, the resulting valid inlet-pressure range is different for each microchannel radius. Therefore, all trends discussed below refer only to the respective validated pressure ranges.

As shown in Figure 7a, increasing the microchannel radius *r* consistently reduces the maximum pressure difference over the complete inlet-pressure range. For each radius, $\Delta P_{\max}$ first increases with inlet pressure, reaches a maximum, and then decreases. The inlet pressure corresponding to the maximum shifts toward lower values as the radius increases. This shift follows from the dependence of the local rarefaction parameter on the product of pressure and channel radius. A larger radius therefore reaches the rarefaction range associated with maximum thermal-transpiration performance at a lower inlet pressure.

The reduction in $\Delta P_{\max}$ is also associated with the variation in the relative macrochannel contribution to the equivalent TPD. Figure 7b shows that the equivalent TPD ratio $\chi_{T}$ increases markedly with the microchannel radius. When *r* is much smaller than *R*, the macrochannel reverse thermal-transpiration contribution becomes weak at moderate and high pressures. In contrast, as *r* approaches *R*, the two channel sections experience increasingly similar rarefaction conditions, and the reverse macrochannel contribution remains substantial. Consequently, the net equivalent TPD decreases with increasing *r*, contributing to the observed reduction in $\Delta P_{\max}$.

The maximum mass flow rate plotted in Figure 7c exhibits a different dependence on the microchannel radius. It first increases with *r*, reaches its highest value at an intermediate radius, and then decreases as the microchannel and macrochannel radii become comparable. Among the investigated cases, the largest mass flow rate is obtained near *r* = 15 μm. This non-monotonic behavior results from two competing effects. Increasing *r* substantially reduces the equivalent flow resistance, as shown in Figure 7d, because the circular-channel geometric factor $AL_{C}=\pi r_{C}^{3}$ present in Equation (12) is proportional to the cube of the channel radius. At the same time, increasing *r* reduces the microchannel contribution to the equivalent TPD and increases the relative reverse contribution of the macrochannel, thereby weakening the net equivalent TPD.

Therefore, the radius that maximizes counter-pressure generation is not the same as that which maximizes mass transport. A small microchannel radius is favorable for counter-pressure generation, whereas an intermediate radius provides a better balance between equivalent TPD and flow resistance for mass-flow output. The precise radius corresponding to the maximum mass flow rate depends on the macrochannel geometry and should not be interpreted as a universal optimum.

### 3.4. Effect of Microchannel Length

**Figure 8:**
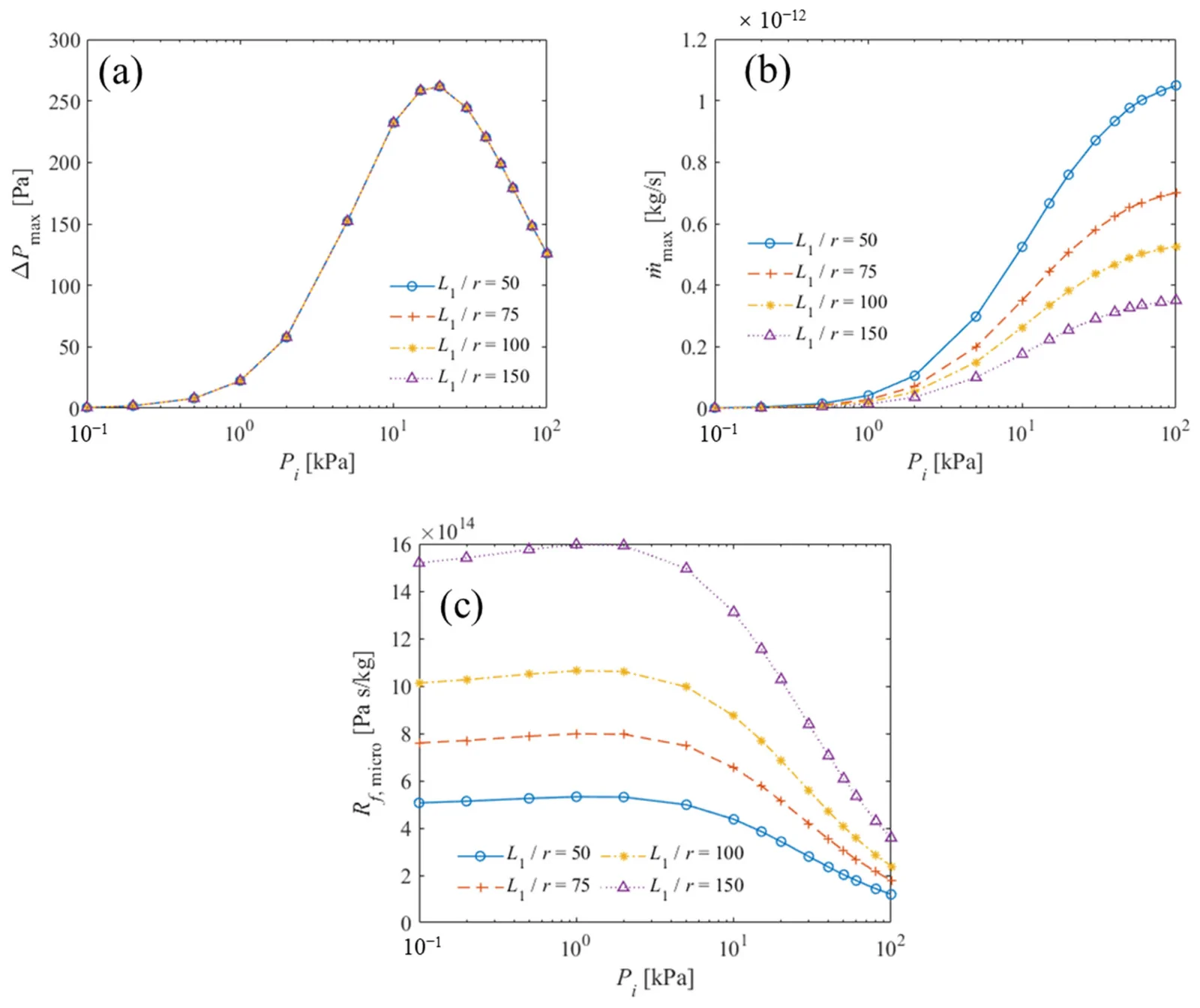
Effects of microchannel length on (**a**) the TPD, (**b**) the maximum mass flow rate, and (**c**) the microchannel equivalent flow resistance.

Figure 8 shows the effects of the microchannel length $L_{1}$ on the TPD, maximum mass flow rate and equivalent microchannel resistance. The length-to-radius ratio was varied from 50 to 150, while the microchannel radius, macrochannel geometry and total temperature difference were kept fixed.

As shown in Figure 8a, the TPD versus inlet pressure curves corresponding to the four microchannel lengths are almost indistinguishable. Both the peak value and the inlet pressure at which the peak occurs remain essentially unchanged. Under the zero-flow condition, increasing $L_{1}$ reduces the local temperature gradient but increases the integration length by the same proportion. Therefore, for fixed temperature endpoints and channel radius, the integrated TPD is nearly independent of the microchannel length under the present conditions.

In contrast, Figure 8b shows that the maximum mass flow rate decreases substantially as $L_{1}$ increases. The relative spacing between the curves indicates an approximately inverse dependence on the microchannel length. This behavior is explained by the equivalent microchannel resistance shown in Figure 8c. For a fixed radius and similar normalized axial distributions, the resistance integral (see Equation (21)) scales approximately with the microchannel length, i.e., $R_{f,micro}\propto L_{1}$. Because the TPD, and therefore the equivalent TPD, remains almost unchanged, the open-system mass flow rate approximately follows ${\dot{m}}_{\max}\propto L_{1}^{-1}$.

The microchannel length therefore has different effects on the two limiting performance measures, $\Delta P_{\max}$ and ${\dot{m}}_{\max}$. It has little influence on the counter-pressure generation but strongly affects the mass-transport capability through the accumulated flow resistance. Shortening the microchannel is consequently effective for increasing the mass-flow output, provided that the long-channel and fully developed-flow assumptions remain valid.

### 3.5. Effect of Macrochannel Radius

**Figure 9:**
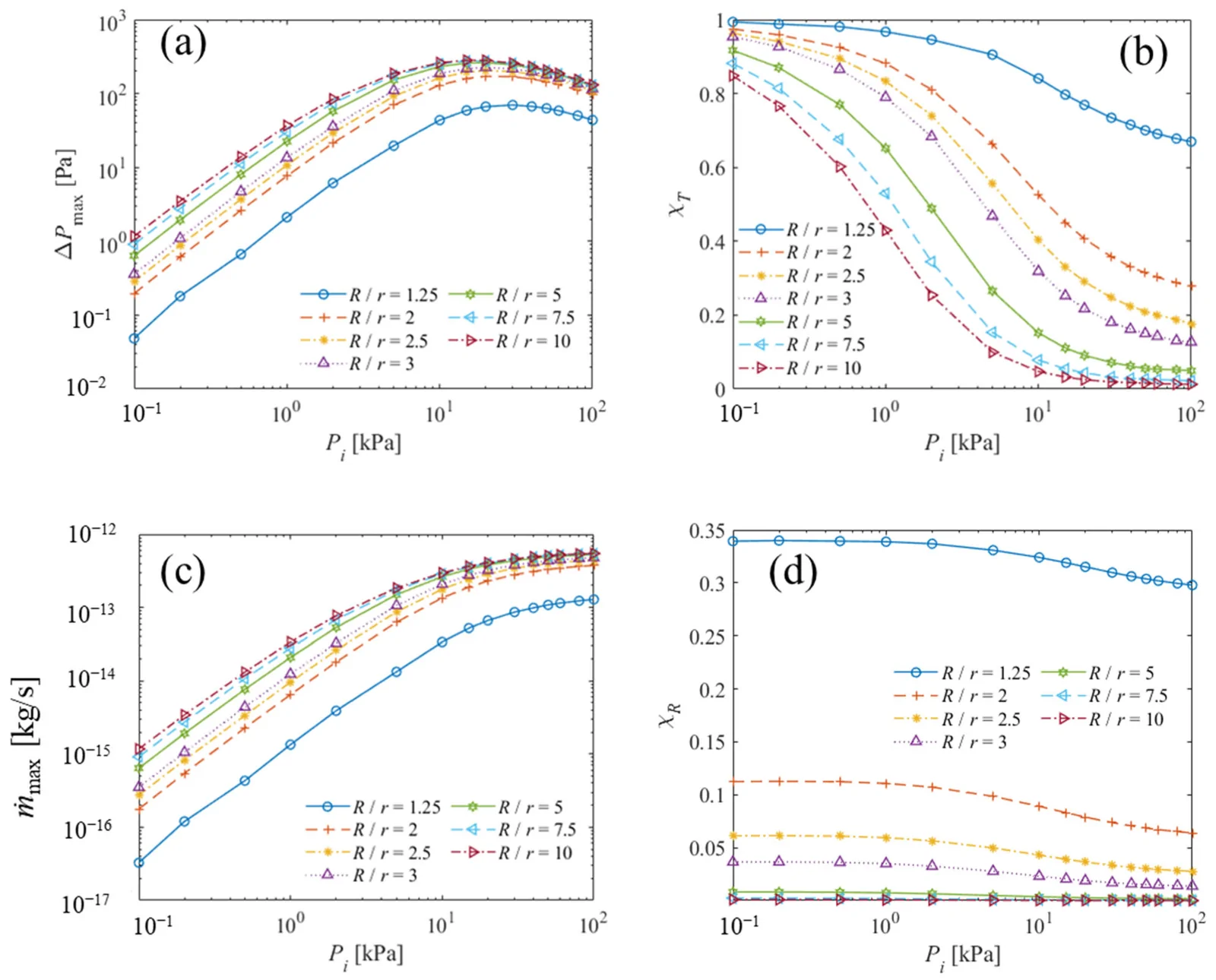
Effects of macrochannel radius on (**a**) the TPD, (**b**) the equivalent TPD ratio, (**c**) the maximum mass flow rate, and (**d**) the equivalent flow resistance ratio.

Figure 9 shows the effects of the macrochannel-to-microchannel radius ratio on the TPD, equivalent TPD ratio, maximum mass flow rate and macrochannel resistance fraction. The microchannel radius and both channel lengths were kept fixed, while the radius ratio R/r was varied from 1.25 to 10.

As shown in Figure 9a, the TPD increases consistently with the macrochannel radius. The improvement is particularly pronounced when R/r increases from 1.25 to approximately 3–5, whereas the curves gradually approach each other for larger radius ratios. Because the TPD equals the equivalent TPD under the zero-flow condition, this increase is primarily associated with the reduction in the magnitude of the reverse thermally driven contribution of the macrochannel.

This interpretation is confirmed by the equivalent TPD ratio plotted in Figure 9b. The ratio $\chi_{T}$ decreases with both inlet pressure and macrochannel radius. When R/r=1.25, the macrochannel and microchannel have comparable radii and therefore experience similar rarefaction conditions, so the reverse macrochannel contribution remains substantial throughout the investigated pressure range. In contrast, for R/r≥7.5, the reverse contribution becomes small at moderate and high pressures. At the lowest pressures, however, a noticeable macrochannel TPD contribution remains even for the largest radius ratios because both channel sections are strongly rarefied.

Figure 9c shows that the maximum mass flow rate also increases with R/r. This enhancement results from two complementary effects: the equivalent TPD increases as the macrochannel thermal-transpiration counteracting effect weakens, while the total flow resistance decreases as the macrochannel conductance increases. The performance gain becomes progressively smaller at large radius ratios, indicating diminishing benefits from further enlargement of the macrochannel.

The resistance decomposition shown in Figure 9d, where the equivalent flow resistance ratio is plotted, provides a quantitative explanation. At R/r=1.25, the macrochannel contributes approximately one-third of the total resistance. Its contribution decreases rapidly with increasing radius ratio and becomes lower than approximately 1% when R/r reaches about 5. Thus, the assumption of negligible macrochannel resistance is valid only for sufficiently large radius ratios and should not be applied to configurations in which the two channel radii are comparable.

Overall, increasing the macrochannel radius improves both pressure-difference generation and mass-flow output by reducing the reverse thermal-transpiration effect and the macrochannel resistance. Under the present geometry, diminishing performance gains become apparent beyond R/r≈5, and the results approach the large-radius limit for R/r≥7.5. Therefore, an excessively large macrochannel radius is unnecessary once both the thermal-transpiration and resistance contributions of the macrochannel have become small.

### 3.6. Effect of Macrochannel Length

**Figure 10:**
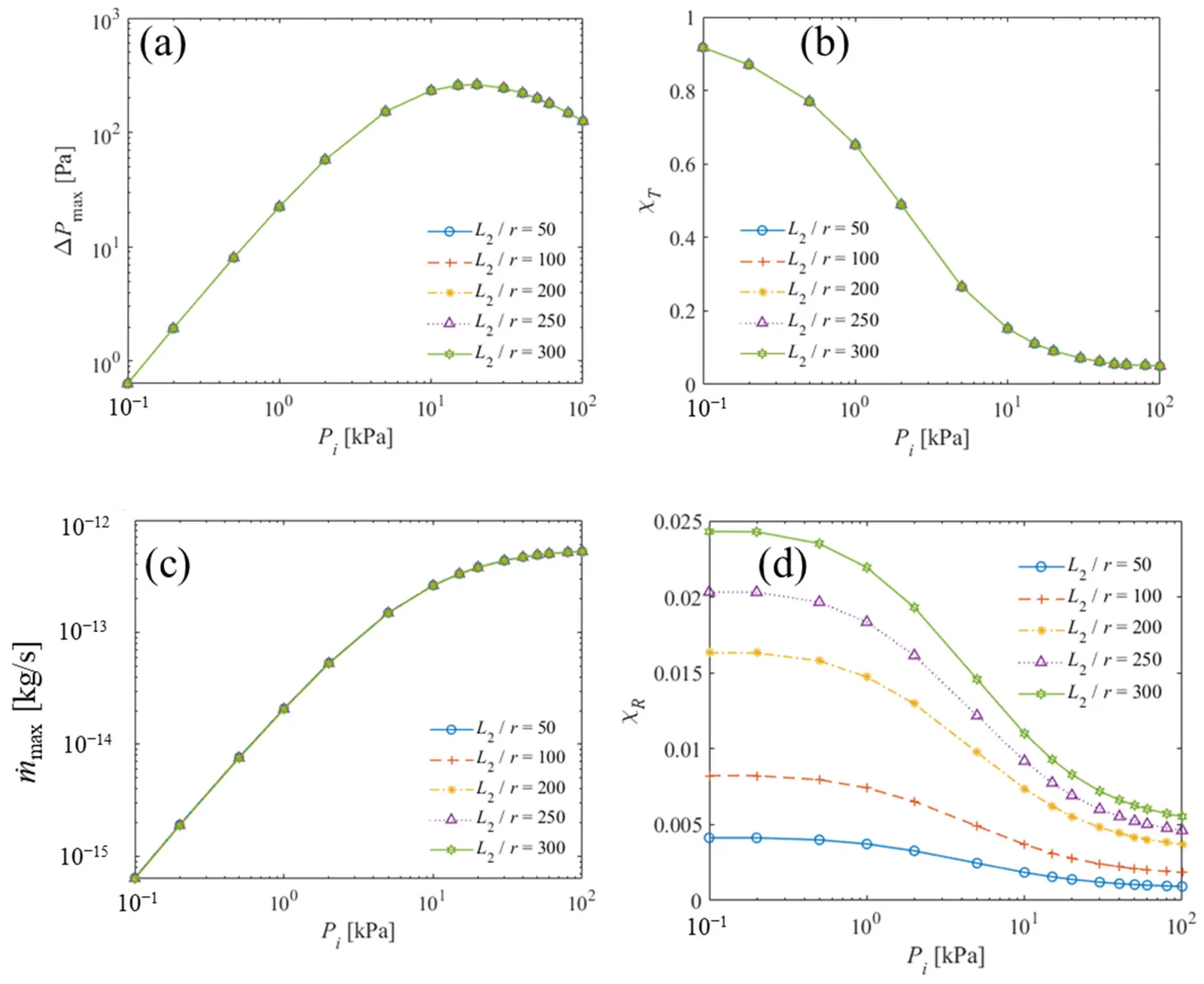
Effects of macrochannel length on (**a**) the TPD, (**b**) the equivalent TPD ratio, (**c**) the maximum mass flow rate, and (**d**) the equivalent flow resistance ratio.

Figure 10 shows the influence of the macrochannel length, $L_{2}$, on the TPD, equivalent TPD ratio, maximum mass flow rate and equivalent flow resistance ratio. The macrochannel length was varied while the two channel radii, microchannel length, and total temperature difference were kept fixed.

As shown in Figure 10a, the TPD curves for all macrochannel lengths are almost indistinguishable. The peak value and the corresponding inlet pressure remain essentially unchanged. Under the zero-flow condition, increasing $L_{2}$ reduces the magnitude of the local macrochannel temperature gradient but increases the integration length by the same proportion. Consequently, for a fixed macrochannel radius and temperature endpoints, the equivalent TPD contribution of the macrochannel (see Equation (19)) is nearly independent of the macrochannel length. This conclusion is consistent with that drawn in Section 3.4 regarding the effect of the microchannel length.

This result is confirmed by Figure 10b, in which the equivalent TPD ratio curves overlap almost completely. Therefore, changing the macrochannel length does not appreciably alter the relative magnitude of the reverse macrochannel thermal effect. The pressure-generation capability is consequently insensitive to $L_{2}$ within the investigated range.

Figure 10c shows that the maximum mass flow rate is also only weakly affected by the macrochannel length. Although the macrochannel equivalent flow resistance increases with $L_{2}$, its contribution to the total equivalent flow resistance remains small. As shown in Figure 10d, the equivalent flow resistance ratio increases with $L_{2}$ but remains below approximately 2.5% even for the longest macrochannel. The total equivalent flow resistance is therefore still controlled primarily by the microchannel, resulting in nearly overlapping maximum-flow-rate curves.

The effects of the two channel lengths are consequently different. Increasing the microchannel length significantly reduces the mass flow rate because the microchannel dominates the total resistance. In contrast, increasing the macrochannel length produces only a small change because the macrochannel remains a minor resistance component. Within the present range, the macrochannel length may therefore be selected primarily based on microfabrication and packaging constraints and thermal-management requirements rather than pressure or flow performance, provided that the fully developed-flow approximation remains applicable.

### 3.7. Quantitative Sensitivity Ranking

**Figure 11:**
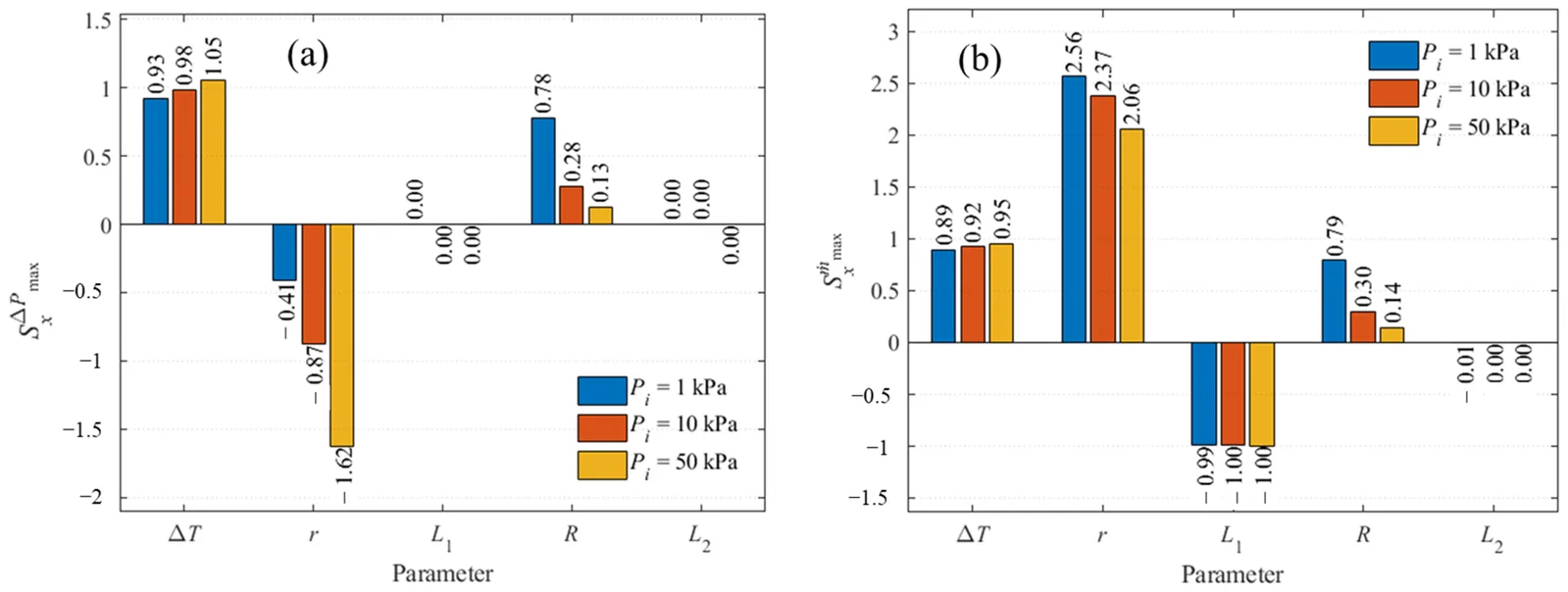
Local logarithmic sensitivities of (**a**) the TPD and (**b**) the maximum mass flow rate at three inlet pressures. Sensitivities were evaluated using centered perturbations of ±10% around the baseline configuration.

Figure 11 presents the local logarithmic sensitivities of the TPD and maximum mass flow rate to the five thermal and geometric parameters. The coefficients were evaluated using centered 10% perturbations around the baseline configuration at inlet pressures of 1, 10 and 50 kPa.

As shown in Figure 11a, the TPD is approximately proportional to the imposed temperature difference ΔT, with $S_{\Delta T}^{\Delta P_{\max}}$ ranging from 0.93 to 1.05. The sensitivity to the microchannel radius *r* is negative, with its magnitude increasing from 0.41 at 1 kPa to 1.62 at 50 kPa, indicating that the radius becomes increasingly important for pressure generation as the inlet pressure increases. In contrast, the positive sensitivity to the macrochannel radius *R* decreases from 0.78 to 0.13, confirming that the reverse macrochannel thermal contribution becomes less important at higher pressures. The two channel lengths have negligible influence on the TPD within the investigated range.

Figure 11b shows a different ranking for the maximum mass flow rate. The microchannel radius has the largest positive local sensitivity, ranging from 2.56 to 2.06. This value reflects the strong increase in channel equivalent flow conductance with radius, partially offset by the simultaneous reduction in the net equivalent TPD. The positive sensitivity is local to the baseline radius and does not imply monotonic growth over the complete radius range, where the mass flow rate exhibits an intermediate-radius maximum.

The microchannel-length sensitivity remains close to −1 at all three pressures, quantitatively confirming the scaling ${\dot{m}}_{\max}\propto L_{1}^{-1}$. The temperature-difference sensitivity ranges from 0.89 to 0.95, indicating a weakly sublinear mass-flow response. The macrochannel-radius sensitivity decreases from 0.79 at 1 kPa to 0.14 at 50 kPa, whereas the macrochannel-length sensitivity remains negligible because the macrochannel contributes only a small fraction of the total resistance.

The sensitivity ranking therefore depends on both the output quantity considered and the inlet pressure. For pressure-difference generation, the temperature difference dominates at low pressure, while the microchannel radius becomes dominant at higher pressure. For mass flow rate, the microchannel radius is consistently the most influential local parameter, followed by the microchannel length and temperature difference. These coefficients describe local engineering responses around the selected baseline geometry and should not be interpreted as universal global scaling exponents.

The sensitivity calculation was repeated using perturbation amplitudes ε=0.05 and 0.20 for the baseline condition. The absolute values of the coefficients changed slightly, but the ranking of the five parameters remained unchanged. The maximum variation in an individual sensitivity coefficient relative to the ε=0.10 result was 3.83%. This confirms that the reported ranking is not an artifact of the selected perturbation interval.

### 3.8. Potential Engineering Applications

The present results are most relevant to microsystems in which low vibration, structural simplicity, and the absence of moving mechanical components are more important than high volumetric throughput. In the closed-system configuration, the circular-channel unit may serve as a basic element for on-chip vacuum conditioning, sealed-cavity pressure adjustment, or low-flow pressure maintenance in MEMS devices. In the open-system configuration, the same unit may be used for carrier-gas circulation or sample transfer in microscale gas-analysis systems, including integrated gas chromatography. Arrays or multistage assemblies of such units may also support thermally driven gas separation and trace-gas sampling.

The two limiting performance metrics, $\Delta P_{\max}$ and ${\dot{m}}_{\max}$, correspond to different application requirements. For systems intended primarily for pressure-difference generation, a smaller microchannel radius and a larger admissible temperature difference generally favor higher pressure differences within the investigated range. Systems intended for gas delivery require a balance between thermal-transpiration driving and equivalent flow resistance and therefore should not be designed solely by minimizing the microchannel radius. The macrochannel should be sufficiently wide to limit reverse thermal transpiration, but increasing its radius beyond the range in which $\chi_{T}$ and $\chi_{R}$ are already small provides limited additional benefit. These applications remain potential design scenarios; the present one-dimensional calculations do not by themselves establish device-level pumping capacity, thermal efficiency, or manufacturability.

### 3.9. Engineering Discussion and Model Limitations

The results demonstrate that the relative importance of the design parameters depends on both the performance metric and inlet pressure. Increasing the temperature difference enhances both the TPD and maximum mass flow rate, with the former varying approximately proportionally to ΔT and the latter showing a weakly sublinear response. The microchannel radius has opposite effects on pressure generation and mass transport: a smaller radius favors the maximum pressure difference, whereas the maximum mass flow rate reaches its highest value at an intermediate radius because the reduction in equivalent flow resistance competes with the decrease in net equivalent TPD. The microchannel length has little influence on $\Delta P_{\max}$, but its equivalent flow resistance increases approximately in proportion to $L_{1}$, resulting in ${\dot{m}}_{\max}\propto L_{1}^{-1}$. Increasing the macrochannel radius improves both outputs by reducing both the reverse thermal-transpiration contribution and the macrochannel equivalent flow resistance, although the gains become progressively smaller at large values of R/r. In contrast, the macrochannel length has only a minor influence within the present range because its resistance remains a small fraction of the total resistance. It may therefore be selected mainly according to microfabrication, integration, thermal-management and packaging requirements.

The present formulation represents each cylindrical channel using locally fully developed circular-tube transport coefficients. It does not resolve the multidimensional interface between the microchannel and macrochannel, entrance and exit regions, finite reservoirs, abrupt expansion losses, temperature jump, wall roughness, fabrication deviations, or conjugate heat transfer. These omissions are expected to affect finite-flow predictions more strongly than zero-flow pressure predictions, although nonequilibrium effects at the interface may influence both quantities. The reported values should therefore be interpreted as quantitative results only within the stated temperature, coefficient, accommodation, and aspect-ratio ranges.

## 4. Conclusions and Outlook

An axial-integration formulation based on transport coefficients of the linearized Shakhov model was used to investigate the transport mechanisms and local parametric sensitivities of a single-stage circular microchannel–macrochannel KµP. The principal quantitative conclusions are as follows.

(1) The pressure-generation and gas-delivery capabilities are controlled by different combinations of TPD and equivalent flow resistance. Under the zero-flow condition, the TPD is equal to the net equivalent TPD, whereas the maximum mass flow rate is governed by the ratio $\Delta P_{T}/R_{f}$. At the baseline condition and $P_{i}=10\;\mathrm{kPa}$, the macrochannel contributes only 0.37% of the total equivalent flow resistance but cancels 15.2% of the positive microchannel equivalent TPD. A channel section that is hydraulically negligible may therefore remain thermally important.
(2) The geometric parameters play distinctly different roles. Increasing the microchannel radius reduces the TPD because it weakens the microchannel thermal-transpiration contribution and increases the relative importance of reverse thermal transpiration in the macrochannel. Its effect on the maximum mass flow rate reflects competition between reduced equivalent flow resistance and reduced net equivalent TPD; consequently, the radii favorable for pressure generation and gas delivery need not coincide. Increasing the microchannel length has little influence on the TPD, but the microchannel resistance scales approximately as $R_{f,micro}\propto L_{1}$, giving ${\dot{m}}_{\max}\propto L_{1}^{-1}$. By contrast, the macrochannel length has only a weak influence within the investigated range because the macrochannel resistance remains a small fraction of the total resistance.
(3) The local sensitivity ranking depends on both the performance metric and the inlet pressure. Over $P_{i}=1\text{–}50\;\mathrm{kPa}$, the value of the temperature-difference sensitivity of $\Delta P_{\max}$ ranges from 0.93 to 1.05, indicating an approximately proportional pressure response. The microchannel-radius sensitivity of $\Delta P_{\max}$ changes from −0.41 to −1.62 as the inlet pressure increases. For ${\dot{m}}_{\max}$, the microchannel radius has the largest local sensitivity, ranging from 2.06 to 2.56, while the microchannel-length sensitivity remains close to −1. The macrochannel-length sensitivity is negligible under the baseline geometric conditions.
(4) Increasing the macrochannel radius improves both the TPD and maximum mass flow rate by reducing reverse thermal transpiration and the macrochannel share of the distributed resistance. The improvement is most pronounced as R/r increases from 1.25 to approximately 3–5 and becomes progressively smaller at larger radius ratios. The macrochannel radius should therefore be selected according to the onset of performance saturation rather than increased without limit.

The quantitative conclusions are restricted to helium, fully diffuse gas–surface reflection, the stated temperature range, and the geometrically admissible long-channel conditions. The principal quantitative temperature range is ΔT=10–50 K; the 75 and 100 K cases provide trend-level information only. Entrance, exit and junction local effects, abrupt expansion or contraction losses, wall roughness, fabrication deviations, temperature jump, and conjugate heat transfer are not resolved. Future work should therefore include multidimensional kinetic or DSMC verification of the microchannel–macrochannel interface, experimental validation under controlled gas–surface accommodation conditions, and extension of the equivalent TPD–equivalent flow resistance framework to parallel and multistage Knudsen-pump systems.

## Figures and Tables

**Table 1:** Thermophysical properties of the working gas and gas–surface interaction model.

Quantity	Symbol	Value
Working gas	–	Helium (He)
Molar mass	*M*	4.0026 × 10^−3^ kg mol^−1^
Specific gas constant	$R_{g}$	2077.1 J kg^−1^ K^−1^
Reference viscosity	$\mu_{ref}$	1.865 × 10^−5^ Pa s
Reference temperature	$T_{ref}$	273.15 K
Viscosity exponent	*ω*	0.66
Tangential momentum accommodation coefficient	*α*	1.0 (diffuse reflection)

**Table 2:** Experimental conditions of Ref. [31] and corresponding conditions adopted in the present benchmark calculation.

Item	Ref. [31]	Present Calculation
Selected dataset	Helium, Δ*T* = 50 K	Same dataset
Channel geometry	Uniform circular glass microtube	Uniform circular microtube
Tube length	30.53 mm	30.53 mm
Internal diameter	490 µm	490 μm
Cold-side temperature	Room temperature; exact value not explicitly specified	292 K, adopted as a representative room temperature
Hot-side temperature	50 K above the cold-side temperature	342 K
Wall-temperature condition	Axial temperature gradient between the cold and hot ends	Linear profile between 292 and 342 K
Operating condition	Final zero-net-flow equilibrium	$\dot{m}=0$
Independent variable	Representative rarefaction parameter	$N_{d}=27$ equivalent inlet pressures obtained using the present helium property relation
Gas–wall accommodation	Not explicitly reported	Fully diffuse reflection, α=1
Compared output	Thermomolecular pressure difference	$\Delta P_{\max}=P_{o}-P_{i}$
Data source	Published curve in Figure 4a	Digitized and converted to Pa

**Table 3:** Parameter matrix for the performance and sensitivity calculations.

Study	Varied Parameter	Values	Fixed Conditions	Validity Indicators
Temperature difference	∆*T*	10, 20, 50, 75, 100 K	Baseline geometry	$\varepsilon_{T}=$ 0.033 − 0.286, cases with ∆*T* ≤ 50 K used quantitatively
Microchannel radius	*r*	2.5, 5, 10, 15, 20, 22.5 μm	*R* = 25 μm; *L*_1_ = *L*_2_ = 500 μm; ∆*T* = 50 K	Report R/r and $L_{1}/r$ for each case
Microchannel length-to-radius ratio	$L_{1}/r$	50, 75, 100, 150	*r* = 5 μm; *R* = 25 μm; *L*_2_ = 500 μm; ∆*T* = 50 K	$L_{1}/r\geq 50$
Radius ratio	R/r	1.25, 2, 2.5, 3, 5, 7.5, 10	*r* = 5 μm; *L*_1_ = *L*_2_ = 500 μm; ∆*T* = 50 K	$L_{2}/R\geq 10$
Macrochannel length-to-radius ratio	$L_{2}/R$	50, 100, 200, 250, 300	*r* = 5 μm; *R* = 25 μm; *L*_1_ = 500 μm; ∆*T* = 50 K	$L_{2}/R\geq 10$
Local sensitivity	±10% about baseline	Five parameters	$P_{i}=1,10,50\;\mathrm{kPa}$	One parameter varied at a time

## Data Availability

The data supporting the findings of this study are available from the corresponding author upon reasonable request.

## References

[1] Wang X., Su T., Zhang W., Zhang Z., Zhang S. (2020). Knudsen pumps: A review. Microsyst. Nanoeng..

[2] Wongwiwat J., Bhuripanyo P., Ronney P. (2019). Hydrocarbon-Fueled Electrical Power Generator with no Moving Parts. Proceedings of the AIAA Scitech 2019 Forum.

[3] Cheng Q., Qin Y., Gianchandani Y.B. (2021). A Bidirectional Knudsen Pump with a 3D-Printed Thermal Management Platform. Micromachines.

[4] Zhao X., Byambadorj T., Qian T., Xu Q., Winship D., Ma Y., Qin Y., Gianchandani Y.B. (2025). Monolithic integration of Knudsen pumps to form a complete, self-sufficient fluidic system for microscale gas chromatography. Microsyst. Nanoeng..

[5] Byambadorj T., Zhao X., Qin Y., Gianchandani Y.B. (2024). Monolithic SOI through-wafer Knudsen pumps with mechanically robust Si channels. Sens. Actuators A Phys..

[6] Basdanis T., Leelaburanathanakul P., Camps T., Rojas-Cárdenas M., Barrot C., Baldas L., Brandner J.J., Colin S. (2026). Knudsen pump for atmospheric and over-atmospheric pressure applications: System design, fabrication procedure and performance evaluation. Appl. Therm. Eng..

[7] An S., Gupta N.K., Gianchandani Y.B. (2014). A Si-Micromachined 162-Stage Two-Part Knudsen Pump for On-Chip Vacuum. J. Microelectromech. Syst..

[8] Kugimoto K., Hirota Y., Kizaki Y., Yamaguchi H., Niimi T. (2017). Performance prediction method for a multi-stage Knudsen pump. Phys. Fluids.

[9] Nakaye S., Sugimoto H. (2016). Demonstration of a gas separator composed of Knudsen pumps. Vacuum.

[10] Sugimoto S., Sugimoto H. (2020). Driving mechanism of thermal transpiration pump with porous material. AIP Adv..

[11] Parittothok P., Poolwech C., Tanteng T., Wongwiwat J. (2022). Performance Improvement of Glass Microfiber Based Thermal Transpiration Pump Using TPMS. Micromachines.

[12] Kosyanchuk V., Petrov A., Kik M., Yagodina M., Seredenko R. (2025). Theoretical and Experimental Study of 3D-Printed Knudsen Pump with Liquid Cooling. Int. J. Heat Mass Transf..

[13] Zhao L., Wang X., Zhang Z. (2023). Numerical investigation into the compression characteristics of a multi-stage Knudsen pump with rectangular channels. Eur. Phys. J. Plus.

[14] Xiao T., Liao Y., Liu X., Pan C. (2025). Numerical investigation of the performance and gas flow characteristics of a novel low-temperature-driven multistage Knudsen pump. Cryogenics.

[15] Zhang Z., Wang X., Zhao L., Zhang S., Zhao F. (2019). Study of Flow Characteristics of Gas Mixtures in a Rectangular Knudsen Pump. Micromachines.

[16] Du C., Wang X., Han F., Ren X., Zhang Z. (2020). Numerical Investigation into the Flow Characteristics of Gas Mixtures in Knudsen Pump with Variable Soft Sphere Model. Micromachines.

[17] Yakunchikov A., Kosyanchuk V. (2019). Numerical investigation of gas separation in the system of filaments with different temperatures. Int. J. Heat Mass Transf..

[18] Shao J., Ye J., Zhang Y., Salem S., Zhao Z., Yu J. (2019). Effect of the microchannel obstacles on the pressure performance and flow behaviors of the hydrogen Knudsen compressor. Int. J. Hydrogen Energy.

[19] Ye J., Shao J., Xie J., Zhao Z., Yu J., Zhang Y., Salem S. (2019). The hydrogen flow characteristics of the multistage hydrogen Knudsen compressor based on the thermal transpiration effect. Int. J. Hydrogen Energy.

[20] Ye J., Jiao X., Tang S., Shao J., Zhao Z. (2021). Three dimensional channel effect on the flow characteristics and the performance of hydrogen Knudsen compressors. Int. J. Hydrogen Energy.

[21] Ye J., Huang Y., Li D., Xie J., He X., Xiao J. (2026). Flow and compression behaviors in hydrogen Knudsen compressors with varying microchannel outlet sizes. Int. J. Heat Fluid Flow.

[22] Lan J., Yang P., Nie W., He Y., Mi L. (2025). Flow behaviors and transmission characteristics of Knudsen pump with blocked microchannels. J. Micromech. Microeng..

[23] Huang Y., Ye J., Li D., Xie J., He X. (2025). Numerical investigation of flow and transmission behaviors in multi-stage hydrogen Knudsen pump with blocked microchannel. Appl. Therm. Eng..

[24] Méolans J.G., Graur I.A. (2008). Continuum analytical modelling of thermal creep. Eur. J. Mech. B/Fluids.

[25] Sharipov F. (1999). Non-isothermal gas flow through rectangular microchannels. J. Micromech. Microeng..

[26] Sharipov F. (1999). Rarefied gas flow through a long rectangular channel. J. Vac. Sci. Technol. A Vac. Surf. Films.

[27] Sharipov F. (1996). Rarefied gas flow through a long tube at any temperature ratio. J. Vac. Sci. Technol. A Vac. Surf. Films.

[28] Sharipov F. (1997). Rarefied gas flow through a long tube at arbitrary pressure and temperature drops. J. Vac. Sci. Technol. A Vac. Surf. Films.

[29] Sharipov F., Bertoldo G. (2005). Rarefied gas flow through a long tube of variable radius. J. Vac. Sci. Technol. A Vac. Surf. Films.

[30] Sharipov F., Seleznev V. (1998). Data on Internal Rarefied Gas Flows. J. Phys. Chem. Ref. Data.

[31] Rojas Cardenas M., Graur I., Perrier P., Meolans J.G. (2011). Thermal transpiration flow: A circular cross-section microtube submitted to a temperature gradient. Phys. Fluids.

[32] Sugimoto S., Sugimoto H. (2022). Quantitative numerical analysis of micro-thermal transpiration pump using kinetic theory of gases. Microfluid. Nanofluid.

[33] Yamaguchi H., Kikugawa G. (2021). Molecular dynamics study on flow structure inside a thermal transpiration flow field. Phys. Fluids.

[34] Tatsios G., Lopez Quesada G., Rojas-Cardenas M., Baldas L., Colin S., Valougeorgis D. (2017). Computational investigation and parametrization of the pumping effect in temperature-driven flows through long tapered channels. Microfluid. Nanofluid.

[35] Rovenskaya O.I. (2020). Numerical investigation of thermally generated gas flow between saw-tooth like surfaces. Int. J. Heat Mass Transf..

[36] López Quesada G., Tatsios G., Valougeorgis D., Rojas-Cárdenas M., Baldas L., Barrot C., Colin S. (2019). Design Guidelines for Thermally Driven Micropumps of Different Architectures Based on Target Applications via Kinetic Modeling and Simulations. Micromachines.

[37] López Quesada G., Tatsios G., Valougeorgis D., Rojas-Cárdenas M., Baldas L., Barrot C., Colin S. (2020). Thermally driven pumps and diodes in multistage assemblies consisting of microchannels with converging, diverging and uniform rectangular cross sections. Microfluid. Nanofluid.

[38] Wang X., Zhang Z., Liu X., Hu P., Han D. (2024). Effects of geometrical parameters on rarefied gas flows and Knudsen force in the system of triangular-rectangular beams with different temperatures. Int. Commun. Heat Mass Transf..

[39] Lekzian E., Sabouri M. (2024). DSMC investigation on rarefied gas mixing through diverging and converging channels. Int. Commun. Heat Mass Transf..

[40] Sazhin O., Sazhin A. (2023). Rarefied gas flow into vacuum through linearly diverging and converging channels. Int. J. Heat Mass Transf..

[41] Zhang D., López-Quesada G., Bergdolt S., Hengsbach S., Bade K., Colin S., Rojas-Cárdenas M. (2023). 3D micro-structures for rarefied gas flow applications manufactured via two-photon-polymerization. Vacuum.

[42] Schweizer F., Bade K., Baldas L., Bergdolt S., Colin S., Deutschbein C., Hengsbach S., Korvink J.G., Rojas-Cárdenas M., Brandner J.J. (2025). Rarefied gas flows in complex microfluidic 3D-structures fabricated via additive manufacturing. Vacuum.

[43] Tantos C. (2019). Polyatomic thermal creep flows through long microchannels at large temperature ratios. J. Vac. Sci. Technol. A Vac. Surf. Films.

[44] Basdanis T., Tatsios G., Valougeorgis D. (2024). Sensitivity analysis of the Cercignani—Lampis accommodation coefficients in prototype rarefied gas flow and heat transfer problems via the Monte Carlo method. Eur. J. Mech. B/Fluids.

[45] Valougeorgis D., Vasileiadis N., Titarev V. (2017). Validity range of linear kinetic modeling in rarefied pressure driven single gas flows through circular capillaries. Eur. J. Mech. B/Fluids.

